# Myocardial metabolic alterations in mice with diet-induced atherosclerosis: linking sulfur amino acid and lipid metabolism

**DOI:** 10.1038/s41598-017-13991-z

**Published:** 2017-10-19

**Authors:** Jueun Lee, Sunhee Jung, Nami Kim, Min-Jeong Shin, Do Hyun Ryu, Geum-Sook Hwang

**Affiliations:** 1 Integrated Metabolomics Research Group, Western Seoul Center, Korea Basic Science Institute, Seoul, 03759 Republic of Korea; 2 Department of Chemistry, Sungkyunkwan University, Suwon, 16419 Republic of Korea; 3 Department of Public Health Sciences, Korea University, Seoul, 02841 Republic of Korea; 4 Department of Chemistry and Nano Science, Ewha Womans University, Seoul, Republic of Korea

## Abstract

Atherosclerosis is a leading cause of cardiovascular disease (CVD), but the effect of diet on the atherosclerotic heart’s metabolism is unclear. We used an integrated metabolomics and lipidomics approach to evaluate metabolic perturbations in heart and serum from mice fed an atherogenic diet (AD) for 8, 16, and 25 weeks. Nuclear magnetic resonance (NMR)-based metabolomics revealed significant changes in sulfur amino acid (SAA) and lipid metabolism in heart from AD mice compared with heart from normal diet mice. Higher SAA levels in AD mice were quantitatively verified using liquid chromatography-mass spectrometry (LC/MS). Lipidomic profiling revealed that fatty acid and triglyceride (TG) levels in the AD group were altered depending on the degree of unsaturation. Additionally, levels of SCD1, SREBP-1, and PPARγ were reduced in AD mice after 25 weeks, while levels of reactive oxygen species were elevated. The results suggest that a long-term AD leads to SAA metabolism dysregulation and increased oxidative stress in the heart, causing SCD1 activity suppression and accumulation of toxic TGs with a low degree of unsaturation. These findings demonstrate that the SAA metabolic pathway is a promising therapeutic target for CVD treatment and that metabolomics can be used to investigate the metabolic signature of atherosclerosis.

## Introduction

Atherosclerosis involves the formation of arterial lesions through the accumulation of cholesterol particles, extracellular matrix deposits, cellular by-products, and inflammatory cells within vessel walls, leading to arterial wall thickening, cracking, or rupture^1^. It is the major cause of cardiovascular disease (CVD), which is drastically increasing worldwide, and contributes substantially to human morbidity and mortality^2^. Therefore, considerable research has been focused on the processes and mechanisms of atherosclerosis, but it is still not fully understood.

A leading cause of atherosclerosis is the overconsumption of high-cholesterol or high-fat diets, which increases blood cholesterol and lipid levels. To elucidate the pathogenesis of atherosclerosis, a mouse model of diet-induced atherosclerosis is used in clinical studies^3,4^. When there is an excessive influx and accumulation of lipids in the endothelium, fatty acid oxidation and storage capacity in blood vessels and organs are overwhelmed, causing abnormal signal transduction, oxidative stress, lipotoxicity, and organ dysfunction^5,6^. Previous studies have reported significant structural and functional alterations accompanied by myocardial metabolic remodeling in heart samples from obese subjects^7,8^.

Metabolomics and lipidomics are powerful tools for analyzing and comprehensively quantifying metabolites and lipid species in biological systems and for examining how diseases such as obesity, atherosclerosis, and diabetes cause molecular changes that lead to CVD^9–12^. Previous studies that used platforms such as nuclear magnetic resonance (NMR) spectroscopy or liquid chromatography–mass spectrometry (LC-MS) reported significant alterations in carnitine, amino acid, fatty acid, choline, and energy metabolism in blood, urine, and liver samples from diet-induced CVD animal models, suggesting that oxidative stress, dyslipidemia, and endothelial dysfunction are associated with atherosclerosis^13–16^. Recently, a multi-platform analytical technique involving NMR and gas chromatography-mass spectrometry (GC-MS) was used to obtain more information on the metabolome in multiple matrices, including biofluids and tissues, altered by atherosclerosis^17^. However, the metabolic changes in a heart with diet-induced atherosclerosis remain unclear.

In this study, an integrated metabolomic and lipidomic analysis was performed using multi-platform analytical tools, including high-resolution magic-angle-spinning (HR-MAS) NMR, solution NMR, and LC-MS, to better understand cardiac metabolism in an atherogenic diet (AD)-induced atherosclerosis mouse model. Our findings may improve the understanding of the complex cardiac pathogenic mechanism of obesity and atherosclerosis.

## Results

### Body weight, heart weight, and serum lipid content

**Figure 1:**
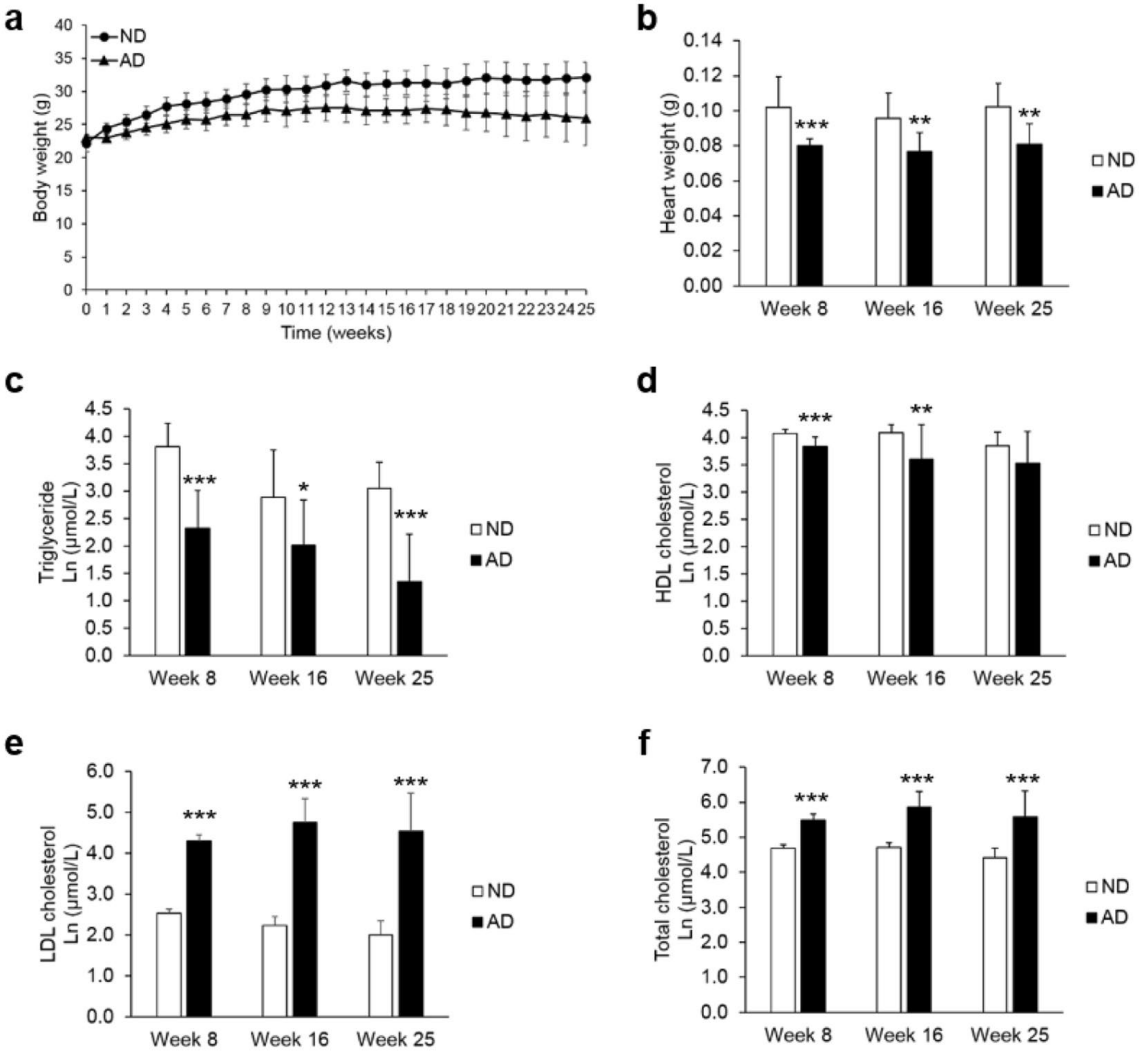
Body weight (**a**), heart weight (**b**) and serum lipid content (**c**–**f**) in mice fed either a normal diet (ND) or an atherogenic diet (AD), as a function of feeding duration. Data represent the mean ± standard deviation. Significant differences between ND and AD mice at weeks 8, 16 and 25 were determined by the Mann-Whitney *U*-test. *^,^ **, and *** indicate *p* < 0.05, *p* < 0.01, *p* < 0.001, respectively.

After AD mice had been fed for 2 weeks, they had a significantly lower average body weight than mice fed a normal diet (ND), and this pattern was consistently observed until week 25 (Fig. 1a: week 8, p < 0.001; week 16, p = 0.001; week 25, p = 0.002). Heart weight was significantly lower in the AD group than in the ND group after 8, 16, and 25 weeks (Fig. 1b). Lower levels of serum triglycerides (TG) and high-density lipoprotein cholesterol (HDL-C) were observed in the AD group than in the ND group. In contrast, higher levels of low-density lipoprotein cholesterol (LDL-C) and total cholesterol (TC) were observed in the AD group than in the ND group after 8, 16, and 25 weeks of feeding (Fig. 1c–f).

### An atherogenic diet causes distinct alterations in myocardial and circulating metabolism

**Figure 2:**
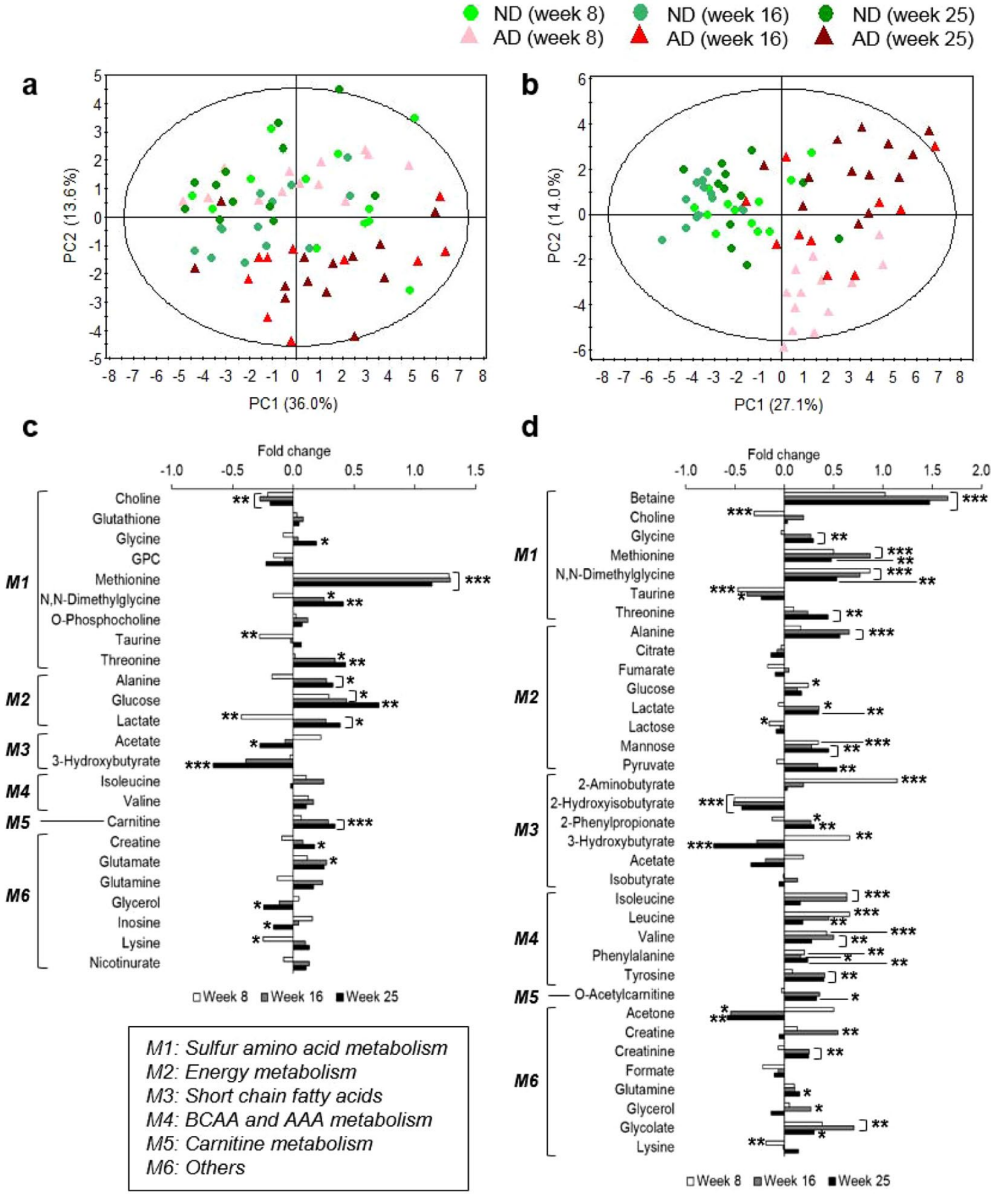
An atherogenic diet (AD) alters myocardial and circulating metabolism. Score plots of principal component analysis (PCA) of heart tissue (**a)** *R* ^2^ *X* = 66.6%, *Q* ^2^ = 36.5%) and serum (**b)** *R* ^2^ *X* = 70.9%, *Q* ^2^ = 32.2%) samples reveals a clear separation between the results from the AD and ND mice. The ellipse represents the 95% confidence region for Hotelling’s T-squared distribution. Relative fold changes in levels of heart (**c**) and serum (**d**) metabolites in AD mice, compared with ND mice, at weeks 8 (white), 16 (gray), and 25 (black). Significant perturbations in sulfur amino acid (SAA), energy, short-chain fatty acid (SCFA), branched-chain amino acid (BCAA), and aromatic amino acid (AAA) metabolism were observed in heart tissue and/or serum. The horizontal axis shows the ratio of metabolic change, (*C*_AD_ − *C*_ND_)/*C*_ND_. *M1* to *M6* indicate arbitrarily assigned metabolisms. Significant differences between ND and AD mice at weeks 8, 16, and 25 were determined by the Mann-Whitney *U*-test. *^,^ **, and *** indicate *p* < 0.05, *p* < 0.01, *p* < 0.001, respectively.

NMR-based targeted metabolic profiling and multivariate and univariate analyses were used to investigate changes in myocardial and circulating metabolism in the AD group. A total of 24 and 35 metabolites from heart tissue (µmol/g) and serum (mmol/L), respectively, were quantified. A principal component analysis (PCA) score plot analyzing serum samples showed a stronger separation between AD and ND groups than that analyzing heart tissue samples. Overall, the PCA plots showed that the separations between ND and AD mice were greatest at week 25 for both heart tissue and serum (Fig. 2). In the PCA score plot analyzing heart tissue samples, the AD mice separated from the ND mice at weeks 16 and 25 (Fig. 2a), and moreover, the AD mice could be easily discriminated from the ND mice at discrete time points (Fig. S1a,c, and e). The PCA score plot analyzing serum samples showed a clear clustering of two groups of mice along the PC 1 axis (Fig. 2b). Additionally, the AD mice were distributed according to the feeding period along PC 2. This pattern was also observed in PCA score plots based on discrete time points (Fig. S1b,d, and f).

The levels of myocardial and circulating metabolites were remarkably changed in AD mice compared to ND mice. In heart tissue of AD mice, SAA, energy, and short-chain fatty acid (SCFA) metabolism were significantly perturbed (Fig. 2c). In particular, these metabolic changes were greater after 25 weeks of feeding than after 8 or 16 weeks of feeding. After 25 weeks of feeding, the AD group, compared to ND mice, had significantly lower levels of choline, acetate, 3-hydroxybutyrate, glycerol, and inosine. In contrast, increased levels of glycine, methionine, N,N-dimethylglycine (DMG), threonine, alanine, glucose, lactate, carnitine, and creatine were observed in the AD group after 25 weeks of feeding.

Furthermore, the AD caused significant metabolic changes in serum as shown by changes in SAA, energy, SCFA, branched-chain amino acid (BCAA), and aromatic amino acid (AAA) metabolism (Fig. 2d). Specifically, levels of serum metabolites such as 2-hydroxyisobutyrate, 3-hydroxybutyrate and acetone were significantly decreased at week 25 in AD mice compared with ND mice. In contrast, higher levels of betaine, glycine, methionine, DMG, threonine, alanine, lactate, mannose, pyruvate, 2-phenylpropionate, valine, phenylalanine, tyrosine, acetylcarnitine, creatinine, glutamine, and glycolate were observed in AD mice than in ND mice. These results indicate that AD alters myocardial and circulating metabolism.

### Sulfur amino acid and lipid myocardial metabolism are affected by an atherogenic diet

**Figure 3:**
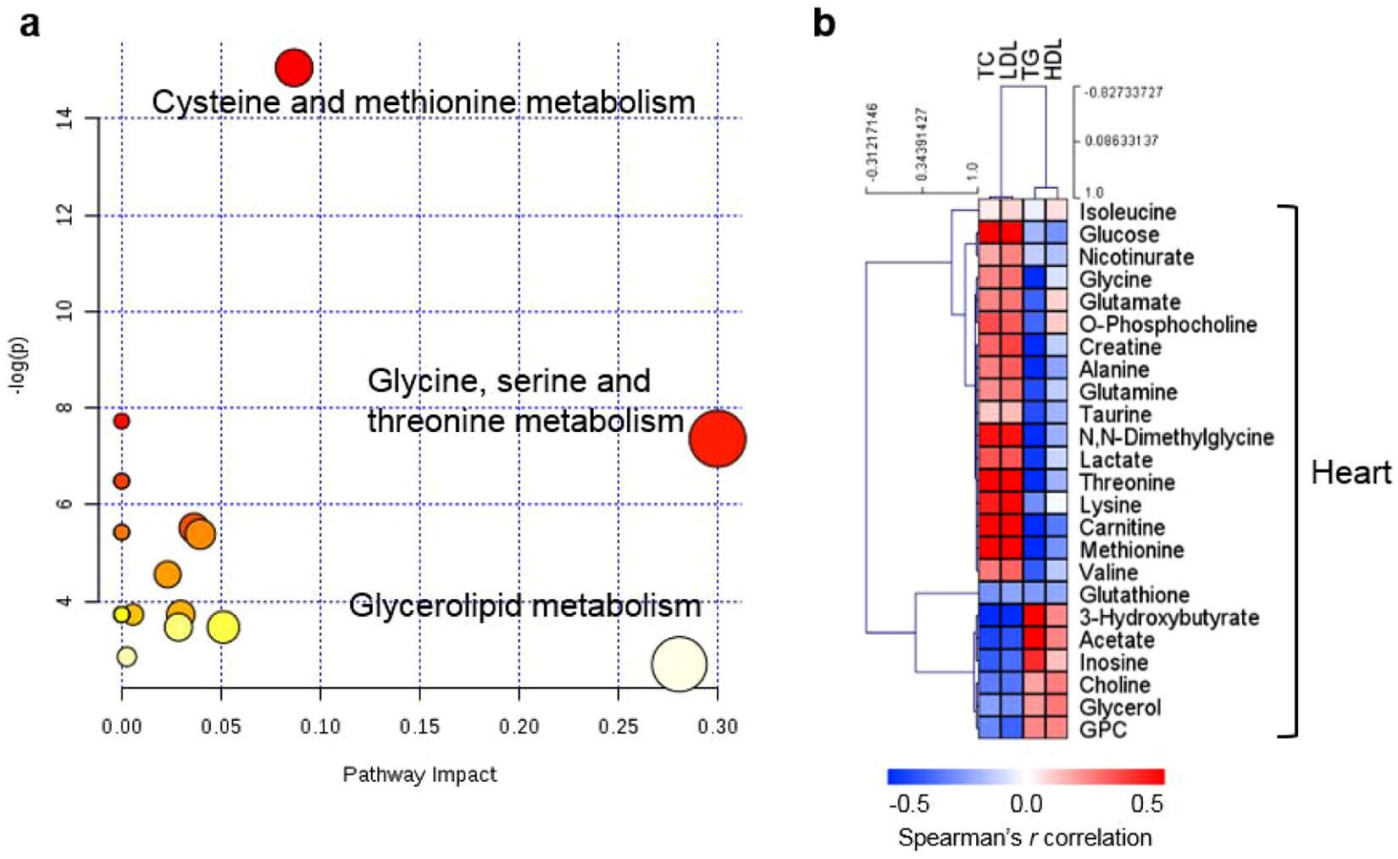
Important heart metabolic pathways influenced by atherogenic diet (AD). (**a**) Overview of pathway analysis of heart tissue from AD and normal diet (ND) mice after 25 weeks. The color and size of the circles reflect the p-values and pathway impact values, respectively. (**b**) Correlation analysis of heart metabolite and serum lipid levels from AD and ND mice after 25 weeks. The correlation matrix shows that serum lipid levels were strongly associated with the levels of metabolites in SAA, lipid, and energy metabolism. Spearman’s correlation coefficients ranged in value from −0.5 to +0.5 and are indicated in red and blue for positive and negative correlations, respectively.

To determine which metabolic pathways change heart metabolism in AD mice, we applied pathway and Spearman’s correlation analyses to metabolic profiles derived from heart samples after 25 weeks of AD; the samples at 25 weeks were selected because metabolic changes were most affected by the AD at this time point (shown in Fig. 2). Based on differential metabolite levels between ND and AD mice after 25 weeks of feeding, a pathway enrichment analysis revealed that the three most enriched metabolic pathways were cysteine and methionine metabolism; glycine, serine, and threonine metabolism; and glycerolipid metabolism (Fig. 3a). Interestingly, pathway enrichment analysis revealed that cysteine and methionine metabolism was one of the key pathways at the other time points (Fig. S2a and c).

Several myocardial metabolites in mice after 25 weeks of feeding were associated with serum lipid content, which is related to the development of atherosclerosis (Fig. 3b). We applied cut-off values to the correlation coefficient (*r* > 0.5 or *r* < −0.5) and p-value (p < 0.05) to identify metabolites that were significantly associated with serum lipid content. Of these metabolites, TGs were most strongly associated with the heart metabolites. HDL levels had no significant relationship with heart metabolite levels. Glucose, threonine, carnitine, and methionine levels were positively correlated with TC and LDL-C levels, whereas 3-hydroxybutyrate content was negatively correlated with lipid content. TG levels were negatively associated with glycine, creatine, alanine, DMG, threonine, carnitine, and methionine levels. In contrast, levels of 3-hydroxybutyrate and acetate were positively associated with TG levels. Additionally, the correlations for the 8-week mice were quite different from those of the 25-week mice (Fig. S2b). However, the correlation patterns for the 16-week mice paralleled those of the 25-week mice (Fig. S2d). These relationships showed that the link between myocardial metabolites and serum lipid content was influenced by the duration of the AD, consistent with that atherosclerosis is a time-dependent disease. These results suggest that SAA and lipid metabolism are important metabolic pathways that are affected by an AD. Thus, we investigated changes in the levels of SAAs and lipid species in heart tissue using liquid chromatography-tandem mass spectrometry (LC-MS/MS).

### Atherogenic diet increases sulfur amino acid metabolite and catabolite levels

**Figure 4:**
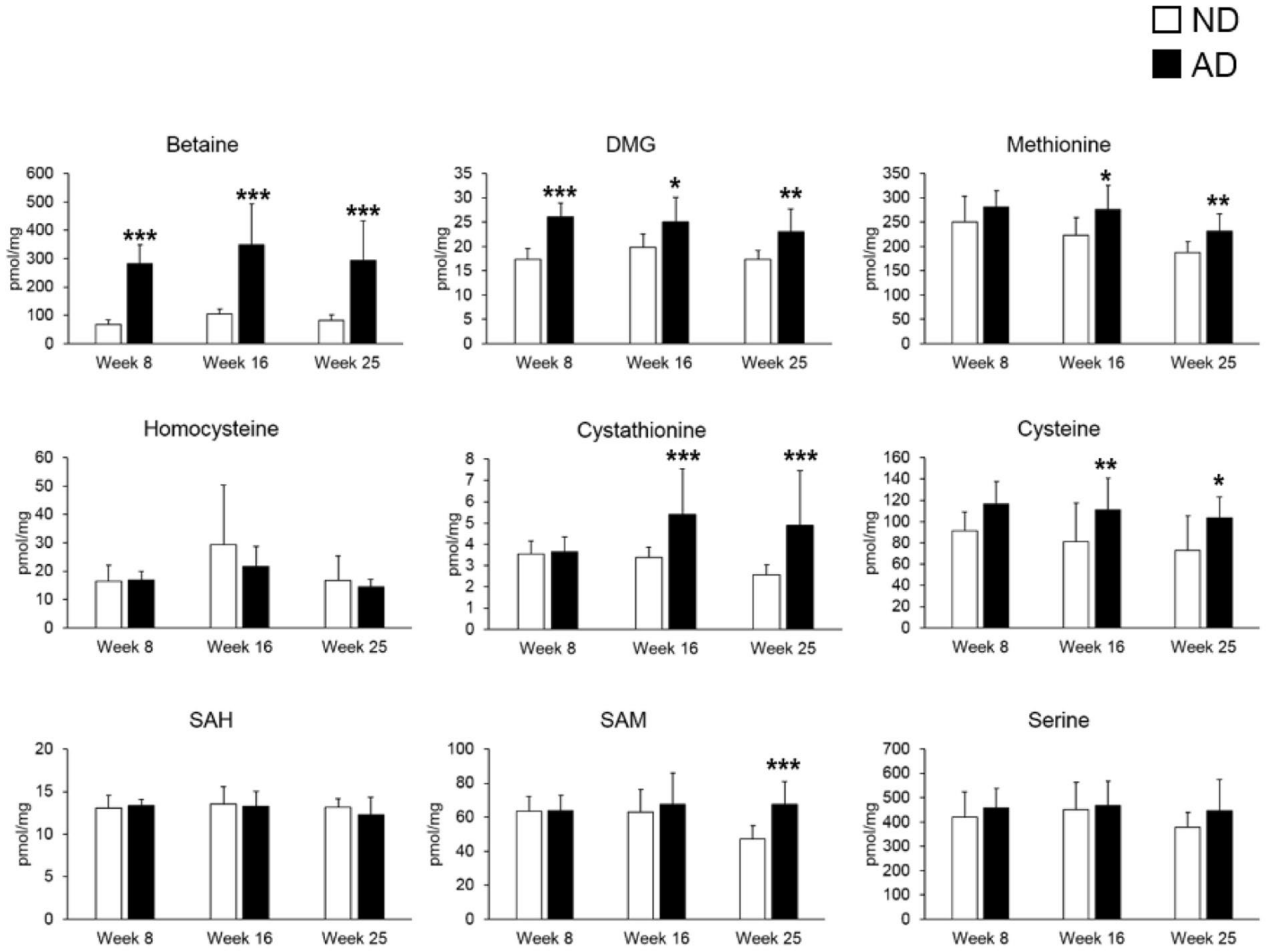
Alterations in the concentrations of sulfur amino acid (SAA) metabolites using LC-MS/MS analysis. SAA metabolites were higher in heart samples from atherogenic diet (AD) mice than normal diet (ND) mice. Concentration is expressed in pmol per mg. Data represent the mean ± standard deviation. Significant differences between ND and AD mice at weeks 8, 16, and 25 were determined by the Mann-Whitney *U*-test. *^,^ **, and *** indicate *p* < 0.05, *p* < 0.01, *p* < 0.001, respectively.

To further verify the changes in the concentrations of SAA metabolites in heart tissue from ND and AD mice, we used LC-MS/MS to quantify the levels of nine metabolites. Representative extract ion chromatograms from heart tissue are shown in Fig. S3. As expected, levels of heart SAA metabolites were significantly changed in AD mice compared with ND mice (Fig. 4). Levels of betaine and DMG were significantly higher in the AD group than the ND group after 8, 16, and 25 weeks of feeding. Levels of methionine, cystathionine, and cysteine were increased in the AD group compared to the ND group after 16 and 25 weeks of feeding. Levels of S-adenosylmethionine (SAM) were higher in AD mice than in ND mice after 25 weeks, but not after 8 and 16 weeks. Identical results were obtained using LC-MS/MS and NMR. These results suggest that increased myocardial SAA metabolism is a distinct characteristic of AD mice.

### Heart tissue from atherogenic diet mice has distinct lipid signatures

**Figure 5:**
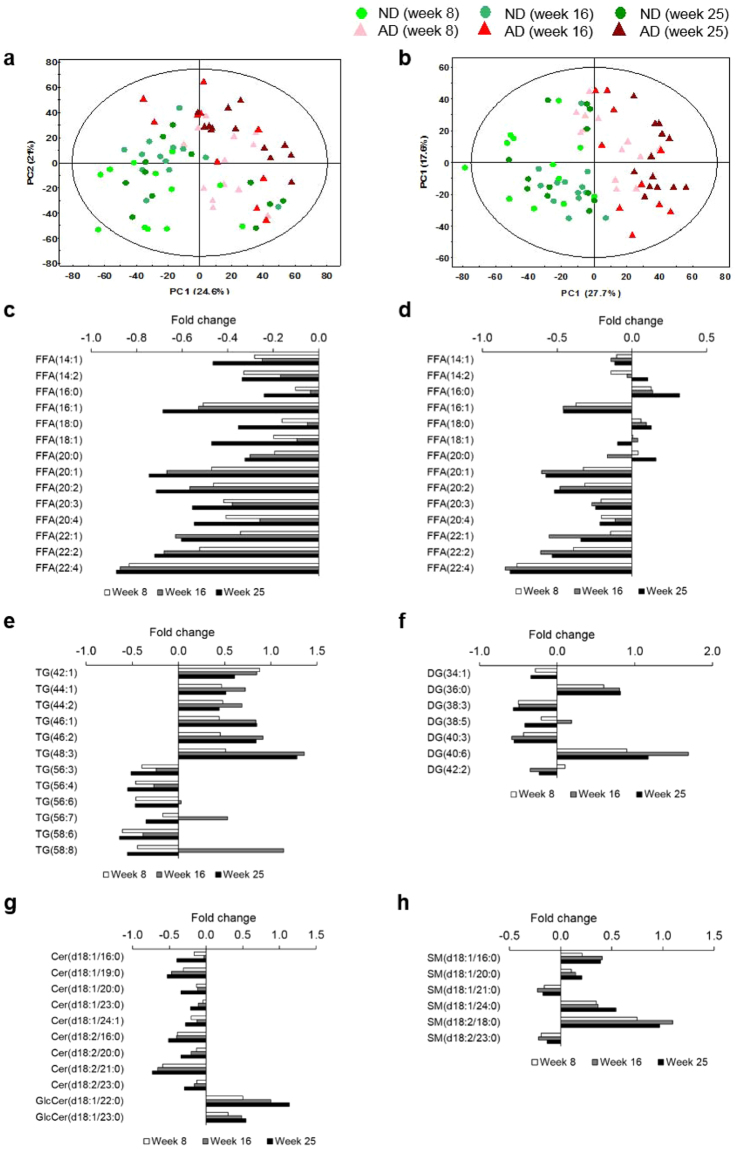
Differential changes in heart lipid species in atherogenic diet (AD) mice. Principle component analysis (PCA) score plots of heart tissues in positive mode ((**a**) *R* ^2^ *X* = 78.3%, *Q* ^2^ = 66.5%) and negative mode ((**b**) *R* ^*2*^ *X* = 78.8%, *Q* ^*2*^ = 67.6%) reveal a clear separation between results from AD mice and normal diet (ND) mice. The ellipse represents the 95% confidence region for Hotelling’s T-squared distribution. Relative fold changes in heart lipid species content in AD compared to ND mice after week 8 (white), 16 (gray), and 25 (black): (**c**) FFAs; (**d**) composition of FFAs; (**e**) TGs; (**f**) DGs; (**g**) Cers; (**h**) SMs. All presented lipid species were significantly altered after 25 weeks of feeding (p < 0.05). The horizontal axis indicates the ratios for lipidomic changes, (*C*_AD_−*C*_ND_)/*C*_ND_. Significant differences between ND and AD mice at weeks 8, 16, and 25 were determined by the Mann-Whitney *U*-test.

A lipidomics approach was used to examine the changes in lipid species in heart tissue from AD mice. PCA score plots showed clear separations between the ND and AD groups along the PC 1 axis in both positive (Fig. 5a) and negative (Fig. 5b) ion modes. PCA score plots analyzing data from heart tissue samples at discrete time points also showed significant separations between AD and ND mice (Fig. S4). Indeed, the separation between the data for the two groups was greatest at week 25. Therefore, we used a variable importance in the projection (VIP) value of >1.0 from partial least squares discriminant analysis (PLS-DA, Fig. S5) and a p-value < 0.05 (calculated using the Mann-Whitney *U*-test on data from samples taken after 25 weeks of feeding) as the selection criteria to identify significant features contributing to lipidomic changes. Table S1 lists the identified lipid species. The significantly different lipid classes that were identified were the ceramides (Cers), glucosylceramides (GlcCers), diacylglycerols (DGs), free fatty acids (FFAs), lysophosphatidylcholines (lysoPCs), lysophosphatidylethanolamines (lysoPEs), phosphatidylcholines (PCs), phosphatidylethanolamines (PEs), sphingomyelins (SMs), and triacylglycerols (TGs).

We observed distinct patterns of lipid species that are involved in the metabolism of FFAs, glycerolipids (TGs and DGs), and sphingolipids (Cers, GlcCers, and SMs). In contrast, lipid species involved in glycerophospholipid metabolism (lysoPCs, lysoPEs, PCs, and PEs) did not have distinct patterns. FFA levels were lower in AD mice than in ND mice at all time points (Fig. 5c). However, in heart tissue, the percentage of saturated fatty acids (SFAs), such as palmitic acid (16:0) and stearic acid (18:0), was significantly higher in AD mice at 25 weeks. Levels of monounsaturated fatty acids (MUFAs) and polyunsaturated fatty acids (PUFAs), on the other hand, were markedly lower (Fig. 5d). Interestingly, heart TG levels showed a clear discriminant pattern based on the degree of unsaturation and the length of the acyl carbon chain (Fig. 5e). TGs that had a low degree of unsaturation and relatively short acyl chains (<48 carbon atoms) were more abundant in AD mice than in ND mice whereas TGs with a high degree of unsaturation and relatively long acyl chains (>56 carbon atoms) were more abundant in ND mice than in AD mice. Next, we examined DG levels and found that DG 36:0, a completely saturated DG, was more abundant in the AD group than the ND group. In contrast, with the exception of DG 40:6, unsaturated DG levels were lower in the AD group than in the ND group (Fig. 5f). All Cer levels were significantly lower in AD mice than in ND mice, whereas GlcCer levels were higher in AD mice than in ND mice (Fig. 5g). With the exception of SM (d18:1/21:0) and SM (d18:2/23:0), levels of SMs with SFA were significantly higher in AD mice than in ND mice (Fig. 5h). These results suggest that in AD mice, the differences in heart lipid levels, such as those of FFAs and TGs, depend on their degree of unsaturation.

### Altered SAA metabolism is associated with lipid metabolism and stearoyl-CoA desaturase-1 activity

We observed the simultaneous perturbation of SAA and lipid metabolism in heart tissue after 25 weeks of AD, which suggests that myocardial metabolism may be changed by a relationship between SAA and lipid metabolism. A previous study found that a disruption in SAA metabolism altered lipoprotein levels in blood^18^ (shown in Fig. 3b). Previous research has also shown that stearoyl-CoA desaturase-1 (SCD1), which produces MUFAs from SFAs, may be a key enzyme linking SAA and lipid metabolism, although the underlying mechanism is poorly understood^19^. Accordingly, we hypothesized that in heart tissue, the accumulation of SAA metabolites is associated with altered lipid metabolism that depends on the degree of unsaturation.

**Figure 6:**
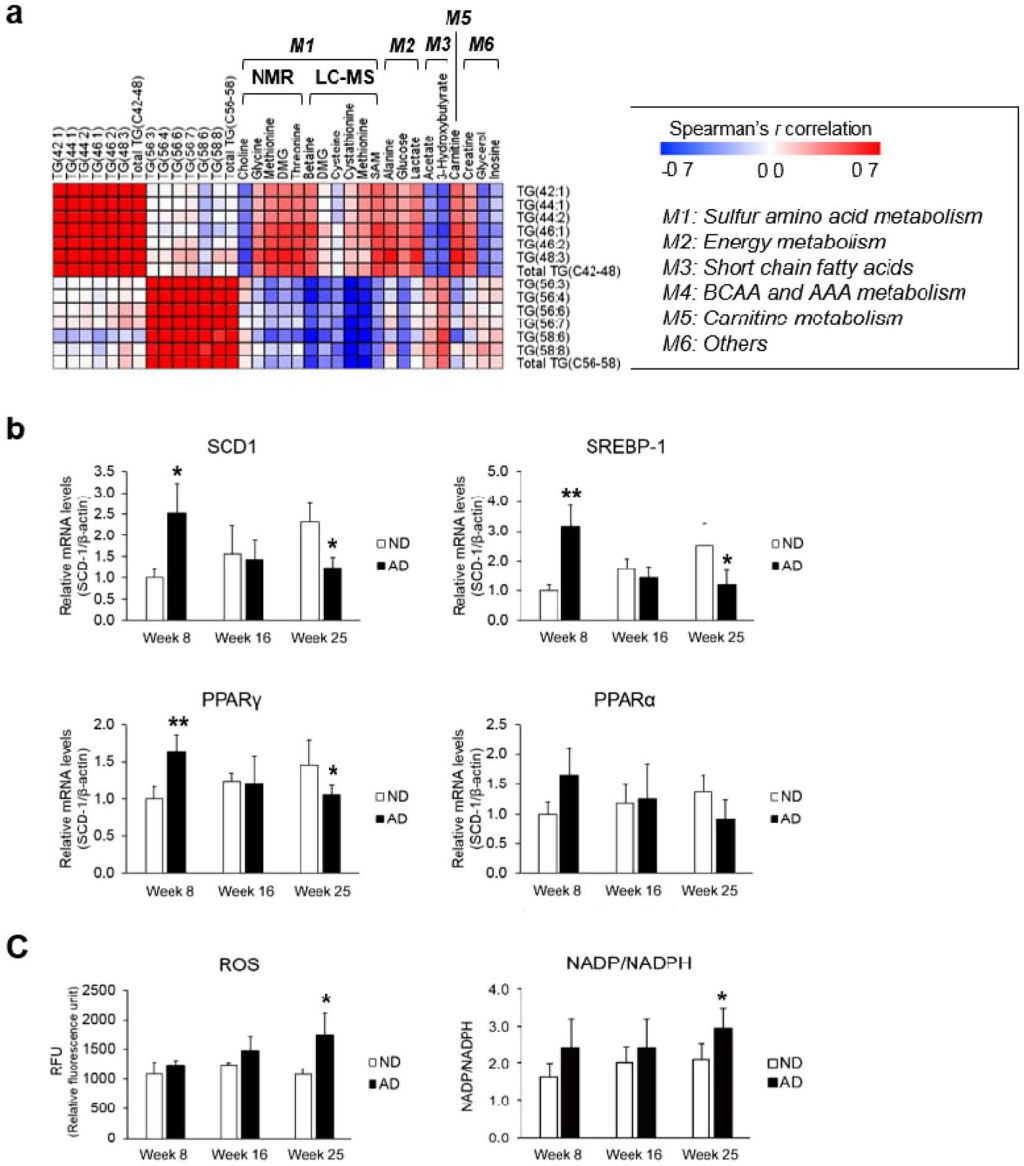
Sulfur amino acid (SAA) and lipid metabolism is linked to SCD1 activity. (**a**) Correlation analysis of polar metabolites and TGs in heart tissue from mice after 25 weeks of normal diet (ND) and atherogenic diet (AD). The relationships between polar metabolites and TGs had different patterns depending on the degree of unsaturation. Spearman’s correlation coefficients ranged in value from −0.7 to +0.7 and are indicated in red and blue for positive and negative correlations, respectively. (**b**) Real-time quantitative PCR analysis showing SCD1, SREBP-1, PPARγ, and PPARα gene expression levels in heart tissues from mice fed ND or AD for 8, 16, and 25 weeks. All gene expression levels were normalized to β-actin mRNA levels as a reference. (**c**) Myocardial reactive oxygen species (ROS) levels are significantly higher after 25 weeks of AD. Relative ROS levels of AD mice, compared with those of ND mice, were estimated by fold changes at week 8. Significant differences between ND and AD mice at weeks 8, 16, and 25 were determined by two-sample *t*-test. *^,^ **, and *** indicate p < 0.05, p < 0.01, p < 0.001, respectively.

Because there were significantly different patterns in the degree of unsaturation for FFAs and TGs, we used Spearman’s correlation analysis to assess the relationship between polar heart metabolites and FFAs and TGs after 25 weeks of AD and ND. TGs with a low degree of unsaturation and relatively short acyl chains (C42–48) had strong positive inter-correlations with each other (0.78 < *r* < 0.97) (Fig. 6a). Additionally, significant positive relationships (0.58 < *r* < 0.97) were observed between TGs with a high degree of unsaturation and relatively long acyl chains (C56–58). Surprisingly, no relationship was found between these two types of TGs, indicating that their metabolic pathways may not be influenced by each other. Intermediates in SAA metabolism had relationships with TGs. In particular, levels of methionine, DMG, threonine, betaine, and SAM were positively correlated, and choline content was negatively correlated with the abundance of TGs with a low degree of unsaturation and relatively short acyl chains. In contrast, methionine, betaine, and cystathionine levels were negatively associated with TGs with a high degree of unsaturation and relatively long acyl chains. TGs and metabolites were significantly related in energy, SCFA, and carnitine metabolism. In the correlation matrix for FFAs, intermediates in SAA, energy and carnitine metabolism showed strong negative associations with FFAs (Fig. S6a). In contrast, SCFA and glycerol levels were positively correlated with FFAs.

The ratio of the product and precursor in the SCD1 enzymatic step, i.e., either a ratio of 16:1 and 16:0 or 18:1 and 18:0, was used to evaluate the effect of AD on SCD1 activity in mouse heart. The levels of two surrogate markers for SCD1 activity were significantly reduced in AD mice (Fig. S6b). Next, we measured SCD1 mRNA expression in heart tissue using real-time quantitative PCR. Unexpectedly, SCD1 mRNA levels were significantly higher in AD mice than in ND mice after 8 weeks of feeding. At weeks 16 and 25, SCD1 mRNA levels in AD mice were the same and significantly lower than in ND mice, respectively (Fig. 6b). Lower TG levels in serum can explain these contrasting results. Plasma TG levels are known to have a positive relationship with the SCD1 desaturation index (16:1/16:0 and 18:1/18:0)^20^. Therefore, the SCD1 desaturation index does not reflect SCD1 activity when circulating TG levels are different between groups. This relationship suggests that SCD1 activity, as determined from the SCD1 desaturation index, should be carefully interpreted^21^.

We also determined the activity of transcription factors in lipid and lipoprotein metabolism in heart tissue. Transcription factors such as SREBP-1, PPARα, and PPARγ regulate the expression of genes such as SCD1 that are involved in lipid and lipoprotein metabolism. SREBP-1 and PPARγ mRNA expression levels showed the same patterns of expression as SCD1 mRNA after 8, 16, and 25 weeks of AD (Fig. 6b). A previous study reported that chronic oxidative stress at a maladaptive stage of atherosclerosis suppressed SCD1 expression and related signaling pathways, leading to induced FA oxidation and apoptosis^22^. Therefore, we next measured ROS levels and the ratio of NADP/NADPH. In heart tissue, ROS levels and the NADP/NADPH ratio were significantly higher in AD mice than in ND mice after 25 weeks (Fig. 6c). These results indicate that altered SAA metabolism in heart from AD mice is associated with lipid metabolism. In addition, long-term AD suppresses SCD1 activity, which regulates the degree of unsaturation of TGs and increases oxidative stress in heart tissue.

## Discussion

In the present study, an integrated metabolomics and lipidomics approach using an NMR- and an LC-MS-based multi-platform enabled us to identify metabolic disturbances in heart tissue and serum. A novel finding was the fact that the AD had a long-term effect on metabolic changes in the heart. To the best of our knowledge, we are the first to report a possible metabolic role of cardiac SAA metabolism in connection to SCD1 and lipid metabolism in mice models of diet-induced obesity and atherosclerosis.

In addition to physical inactivity, a high-fat diet is a major risk factor for obesity and atherosclerosis. Thus, AD, being high in cholesterol and cholate, is commonly used in atherosclerosis studies^23,24^. In this work, an AD-induced murine model was used to mimic the Western diet and study the atherogenic process. As a result of the reduced body and heart weight of members of the AD group, we anticipated finding growth inhibition and toxic effects in their heart tissue^25^. We also found that AD caused drastic changes in the levels of serum lipoproteins, which was consistent with findings from previous studies^14,17^. In serum samples from the AD group, LDL-C and TC levels were significantly higher and HDL-C levels were lower than in the ND group, indicating that the AD mice had a higher risk of developing atherosclerosis^26^ and disorders in fatty acid metabolism. The reduced levels of serum TGs were probably due to the high amount of cholesterol and cholate in the diet^27^.

Diet is well known to play a role in the regulation of metabolic pathways in humans^12^. Using murine models of atherosclerosis, previous studies have reported significant changes in blood, urine and liver metabolite content, but only few changes in the heart metabolites^14,17,28^. In this study, however, drastic metabolic changes were observed not only in serum but also in heart tissue samples from AD mice. These changes became more apparent as the duration of the AD increased, indicating that AD has a long-term effect on heart metabolism. Previous studies only evaluated the short-term effects of AD. The chronic imbalance between uptake and utilization of fatty acids in myocardial cells can lead to a very high concentration of fatty acids in the myocardium and eventually overwhelm its capacity to regulate fatty acid metabolism^6,29^. Thus, long-lasting metabolic imbalances may cause changes of greater intensity in myocardial metabolic pathways, leading to myocardial lipotoxicity and dysfunction, than brief metabolic imbalances^30–32^.

In this study, the AD significantly altered SAA metabolism, consistent with results from previous studies on plasma, serum, urine, and liver samples^14–16,33^. Our findings also suggest that SAA metabolism is related to lipid metabolism. Enhanced oxidative stress and suppression of SCD1 and related signaling pathways were caused by the long-term AD. AD mice had significantly higher levels of methionine, SAM, cysteine, cystathionine, betaine, and DMG in their heart tissue. The up-regulated SAA metabolism indicates an increase in activity in the transmethylation-transsulfuration pathway^34,35^, forcing the maintenance of the methionine cycle^36^ and the synthesis of cysteine from methionine. Cysteine is required to produce coenzyme A (CoA-SH), which plays a key role in the oxidation and synthesis of fatty acids^37^ and is a precursor to taurine and glutathione. The levels of taurine and glutathione remained unchanged in heart tissue in AD mice. Thus, the high levels of cysteine in heart from AD mice may be used to produce CoA-SH, leading to chronic oxidative stress.

Interestingly, the relationship between SAA and lipid metabolism has been previously reported. Circulating levels of lipoprotein can be changed by disrupting SAA metabolism^18^. Methionine supplements induce hypercholesterolemia and perturb cholesterol metabolism in the liver^38^. El-Khairy *et al*. reported that plasma total cysteine was a risk factor for vascular disease^39^. In particular, SAA disorder was affected by SCD1 activity^40,41^. SCD1 regulates the degree of unsaturation by converting SFAs to MUFAs and mediates fatty acid metabolism through SREBP-1 and PPARγ, transcription factors involved in the regulation of SCD1 gene expression. Therefore, SCD1 may link SAA and lipid metabolism^19^. However, the mechanism of this process remains to be elucidated.

Obesity and atherosclerosis were previously found to be associated with high SCD1 expression in liver or serum^42^. However, several studies have reported inconsistent findings based on results with atherosclerosis models^43^. For example, Pawel *et al*. reported that loss of the SCD1 gene improved cardiac function in ob/ob mice^44^, whereas another study found that SCD1-deficient mice developed larger atherosclerotic lesions despite reduced plasma TG levels^45^. Additionally, a Western-like diet significantly reduced levels of hepatic SCD1 in LDLR^−/−^ mice^17^. In this study, AD elevated levels of SCD1, SREBP-1, and PPARγ at an early stage. However, the results were reversed when the duration of the AD increased (Fig. 6b). This finding indicates that high levels of SCD1 expression and the corresponding signaling pathways inhibited fatty acid oxidation and apoptosis at an adaptive stage. In contrast, chronic oxidative stress at a maladaptive stage inhibited SCD1 expression and its related signaling pathways, which eventually induced FA oxidation and apoptosis^22^. Thus, enhanced myocardial SAA metabolism can be related to chronic oxidative stress and deactivation of SCD1, thereby altering lipid metabolism.

When SCD1 is deficient, SFAs cannot be converted into MUFAs at a maladaptive stage. As a result, the SFAs accumulated in heart tissue were used to produce TGs with a low degree of unsaturation, rather than those with a high degree of unsaturation. This result is well described and correlated in the two types of TGs (Fig. 6a). In other words, these two types of TGs were synthesized using two different pathways, neither of which involved the conversion of SFAs into MUFAs. In a human study, the accumulation of cardiac TGs was observed in individuals with impaired glucose tolerance and type 2 diabetes, suggesting that high cardiac TG levels are strongly associated with obesity/type 2 diabetes-associated myocardial dysfunction^46,47^. However, it was unclear which TG subspecies contributed to the cardiac pathophysiology. TGs with a low degree of unsaturation are less fluid than TGs with a high degree of unsaturation and can lead to insulin resistance, dysregulated glucose tolerance, cell stress, and even apoptosis^48^. This relationship means that TGs with a low degree of unsaturation are more harmful to myocardial cells in heart tissue than TGs with a high degree of unsaturation.

Dysregulation or inflexibility of energy metabolism is associated with CVDs, including ischemic heart disease, diabetic and obesity cardiac dysfunction, and heart failure^49^. We found that AD caused significant perturbations in glucose metabolism related to myocardial energetics. First, there was a remarkable accumulation of glucose in heart and serum. Fatty acids and glucose are both major substrates used to generate energy in the heart. Because the heart can adapt to changes in the environment by switching from one substrate to another, the high concentration of fatty acids inhibited the utilization of glucose to generate ATP^50^. Previous studies have suggested that a high-fat diet leads to impaired glucose tolerance and reduced insulin sensitivity^51^. Therefore, augmented levels of glucose can be associated with insulin resistance and diabetes in atherosclerosis. Furthermore, we found that AD significantly elevated pyruvate, lactate, and alanine levels but did not change the levels of TCA cycle metabolites such as citrate and fumarate. This result indicates that the inefficient ATP-generating anaerobic glycolysis cycle is employed instead of the TCA cycle in ischemic heart. Previously, hypoxia-induced ATP depletion and lactate accumulation were found to promote atherosclerotic lesion progression^52^. Moreover, an accumulation of lactate can lead to an acidic environment and cause heart toxicity^53^. Increased levels of alanine from glycolysis can be transported to the liver and used for gluconeogenesis to produce glucose; regenerated glucose can then be absorbed by muscle tissue, such as the heart.

AD mice had remarkably low levels of SCFAs and ketone bodies, such as acetate, 3-hydroxybutyrate, 2-hydroxyisobutyrate, and acetone, in serum and heart tissue. Several previous studies have suggested that dietary changes and obesity are strongly related to the diversity and composition of the gut microbiome, which generates key products such as acetate (C_2_), propionate (C_3_), and butyrate (C_4_) through bacterial anaerobic fermentation^54,55^. As demonstrated in animal models, SCFAs, which were transported from the intestine into blood and tissue, improved insulin sensitivity and prevented metabolic diseases such as obesity and diabetes^56–58^. This result is consistent with the functions of SCFAs, which include inhibiting hepatic cholesterol and/or TGs and delaying micronutrient absorption, resulting in increased insulin sensitivity^59^. Therefore, a reduction in SCFA levels in AD mice may lead to the development of atherosclerosis by disturbing lipid metabolism and insulin sensitivity.

In addition, surplus fatty acid availability enhances fatty acid oxidation to produce ATP and its by-products, ketone bodies, instead of glucose oxidation^60^. The produced ketone bodies can be substrates in energy synthesis in myocardium. Increased ketone body oxidation can thus decrease the levels of ketone bodies in heart and serum of mice exposed to an AD.

A high level of circulating BCAAs and AAAs has been reported to be a risk factor for the development of obesity and diabetes with insulin resistance^61–63^. In the present study, we found that BCAA and AAA metabolites were significantly higher in serum from mice fed AD, consistent with previous observations in plasma, serum, kidney, and liver samples^15–17^. In particular, increased BCAA and glucose levels in serum, as well as dysregulated glucose metabolism in heart, are caused by BCAAs inhibiting insulin signaling and the impairment of glucose metabolism in skeletal muscle, which leads to hyperglycemia^64,65^.

Carnitine plays an important role in lipid metabolism because it shuttles long-chain fatty acids from the inside to the outside of the mitochondrial membrane in the process of fatty acid oxidation by carnitine palmitoyltransferase (CTP)^66,67^. It was previously reported that carnitine in red meat promoted the development of atherosclerosis^68^. Additionally, carnitine and CTP deficiencies disrupt fatty acid oxidation and are associated with heart failure^69^. In the current study, we observed elevated levels of carnitine and acetylcarnitine in heart and serum from mice fed ADs. These results suggest that in an atherosclerosis background, increased fatty acid uptake and CTP activity lead to up-regulation in fatty acid oxidation^70^.

## Conclusion

Using metabolic and lipidomic profiling that integrated NMR and LC-MS, we showed that an AD causes significant metabolic perturbations in heart and serum samples from a mouse model of atherosclerosis. Chronic exposure to AD-induced dysregulation of SAA metabolism and excessive oxidative stress in the heart led to SCD1 activity suppression and altered lipid metabolism, as evidenced by an accumulation of toxic TGs with a low degree of unsaturation. Thus, SAA metabolism may be a target for therapeutic exploitation to treat chronic diseases including obesity, dyslipidemia, atherosclerosis, and diabetes. However, further studies are required to reinforce the importance of these findings and to fully clarify the underlying mechanisms. Additionally, we found altered energy, SCFA, BCAA, AAA, and carnitine metabolism in heart and/or serum samples of AD mice. This study provides fundamental information for the future study of atherosclerosis and reinforces the importance of metabolic control through diet management in patients with obesity and early atherosclerosis.

## Materials and Methods

### Animals and sample collection

Five-week-old male C57BL/6 J mice were housed two per cage under a constant temperature of 18–24 °C and a relative humidity of 50–60%. After a 7-day adaptation period, the mice were randomly divided into ND (n = 38) and AD (n = 36) groups. Based on the AIN-76 diet, the AD was modified by the addition of 1.25% (w/w) cholesterol and 0.5% cholate (w/w)^23^. Table S2 lists the complete diet composition. All animals were allowed access to water and feed ad libitum. Body weight was measured once per week. After 8, 16, and 25 weeks of feeding, animals were fasted for 12 h and euthanized to collect sera and hearts, which were stored at −80 °C until use. At least 10 mice (n = 10–13) were included in each group. Heart weight was measured immediately after sacrifice. All studies were performed in accordance with the protocols for animal studies approved by the Committee on Animal Experimentation and Ethics of Korea University (KUIACUC-2013-236).

Serum levels of TC, LDL-C, HDL-C, and TG were determined at the Korea Animal Medical Science Institute.

### Metabolomic and lipidomic analysis based on NMR and LC-MS

High-resolution magic-angle-spinning NMR (Bruker BioSpin, Germany) and solution NMR (Bruker BioSpin) were used for ^1^H NMR-based metabolomic analysis of heart and serum samples, respectively. An Agilent 1290 Infinity LC and 6490 Triple Quadrupole MS system (Agilent Technologies, USA) was used to determine the concentration of SAAs. LC-ESI-MS/MS analysis for lipidomics was performed on a triple TOF™ 5600 MS/MS System (AB Sciex, Canada) combined with a UPLC system (Waters, USA). For further details, see Supplementary Information.

### Real-time quantitative PCR

Total RNA was extracted from 10 mg of heart tissue using an RNeasy Mini Kit (Qiagen, USA) according to the manufacturer’s protocol. Four heart tissues per group were analyzed. RNA concentration and quality were immediately determined using a Nanodrop 2000 (Thermo Fisher Scientific, USA), and aliquots of the total RNA were stored at −80 °C until use. Total RNA was reverse-transcribed using a GoScript Reverse Transcription System (Promega, USA). The cDNA was amplified using GoTaq qPCR master mix according to the manufacturer’s instructions (Promega, USA). Reactions were carried out with SYBR green for 40 cycles (denaturation at 95 °C for 10 s, annealing at 60 °C for 30 s, and extension at 72 °C for 30 s) using the StepOnePlus Real-Time PCR System (Applied Biosystems, USA). The primers used for qPCR are listed in the Supplementary Information. Gene expression was normalized to the mRNA expression level of β-actin (endogenous control), and fold changes were calculated compared to ND mice at week 8 using relative quantification.

### Reactive oxygen species (ROS) and NADP/NADPH analysis

ROS levels in heart tissue were assessed using an OxiSelect ROS/RNS Assay kit (Cell Biolabs, USA) according to the manufacturer’s instructions. Four samples of heart tissue per group were used. The relative fluorescence of the samples was measured using 485-nm excitation/535-nm emission with a microplate reader (Tecan, Austria). ROS levels were determined by comparing the fluorescence of the samples with a 2′,7′-dichlorodihydrofluorescein standard curve. Relative ROS levels of AD mice, compared with those of ND mice, were estimated by fold changes at week 8.

The ratio of NADP/NADPH in heart tissue was assessed using an NADP/NADPH Assay kit (Abcam, UK) according to the manufacturer’s instructions. Five or six tissues per group were used. NADPH and NADP levels were quantified by measuring the OD at 450 nm using a microplate reader (Tecan) and comparing this value with an NADPH standard curve.

### Statistical analysis

SIMCA-P + version 12.0 (Umetrics, Sweden) was used to conduct multivariate data analysis. All metabolite levels were scaled to unit-variance prior to PCA and PLS-DA. PCA was applied to provide an overview of metabolic data. The model validity was assessed by considering model parameters: *R* ^2^ for the interpretability of the model and *Q* ^2^ for the predictability of the model. The Statistical Package for Social Sciences software, version 15.0 (SPSS Inc., USA) and GraphPad Prism, version 7.0a (GraphPad Software, Inc., USA) were used to assess whether each variable was normally distributed by using the Shapiro-Wilk normality test, and statistical significance was assessed using the two-sample *t*-test or Mann–Whitney *U*-test and Spearman’s correlation analysis. Pathway analysis was performed with MetaboAnalyst, a web-based inference of functions and pathways of metabolic data^71^.

### Data availability

NMR and LC-MS Metabolomic data that support the findings of this study have been deposited in MetaboLights (http://www.ebi.ac.uk/metabolights) under the accession codes MTBLS539. All other data are available within the manuscript or from the corresponding authors upon reasonable request. The authors declare that all the data supporting the findings of this study are available within the article and its Supplementary information files and from the corresponding author upon reasonable request.

## Electronic supplementary material

Supplementary Information
