# Supplementary material for: Myocardial metabolic alterations in mice with diet-induced atherosclerosis: linking sulfur amino acid and lipid metabolism
Source: Sci Rep. 2017 Oct 19;7:13597. doi: 10.1038/s41598-017-13991-z (PMC5648757; doi:10.1038/s41598-017-13991-z)
Supplement: Supplementary file 1 — Supplementary Information [file 41598_2017_13991_MOESM1_ESM.pdf]

Supplementary Information

Myocardial metabolic alterations in mice with diet-induced atherosclerosis: linking sulfur amino acid and lipid metabolism

Jueun Lee[1,2,5], Sunhee Jung[1,2,5], Nami Kim[1], Min-Jeong Shin[3], Do Hyun Ryu[2], Geum-Sook Hwang[1,4]

[1]Integrated Metabolomics Research Group, Western Seoul Center, Korea Basic Science Institute, Seoul 03759, Republic of Korea.

[2]Department of Chemistry, Sungkyunkwan University, Suwon 16419, Republic of Korea.

[3]Department of Public Health Sciences, Korea University, Seoul 02841, Republic of Korea

[4]Department of Chemistry and Nano Science, Ewha Womans University, Seoul, Republic of Korea

[5]These authors contributed equally to this work.

Corresponding author

Do Hyun Ryu: Sungkyunkwan University, Suwon 16419, Republic of Korea. Phone: +82 31 290 5931. Fax: +82 31 290 5967. E-mail: dhryu@skku.edu

Geum-Sook Hwang: Korea Basic Science Institute, Seoul 120-140, Republic of Korea. Phone: +82-2-6908-6200. Fax: +82-2-6908-6239. E-mail: gshwang@kbsi.re.kr

^{1}H NMR-based metabolomic analysis

For serum samples, 90 μL of serum was mixed with 510 μL of saline solution (0.9% w/v sodium chloride in deuterium oxide) and transferred to 5-mm NMR tubes. One-dimensional ^{1}H NMR spectra of serum were acquired at 298 K on a Bruker Avance III HD 800 MHz NMR spectrometer (Bruker BioSpin, Germany) with a Bruker 5 mm CPTCI Z-GRD probe. The water-suppressed CPMG spin-echo pulse sequence (RD-90˚-[τ-180˚- τ] n-ACQ) was used to attenuate broad signals from proteins and lipoproteins with total T_2 filter time of 32 ms. For all spectra of each serum sample, 128 transients were acquired with 64 k data points, a spectral width of 16,025.641 Hz, and the relaxation delay of 4 s, and acquisition time of 2.045 s.

For heart tissues, 10-12mg of each sample was transferred into a disposable insert tube. The residual space in the tube was filled with deuterium oxide (99.9 atom % D). And then, the disposable insert tube was inserted into a zirconium HR-MAS rotor (4mm outer diameter). The samples were maintained in a refrigerated tray at - 20˚C before the analysis. All HR-MAS ^{1}H NMR spectra of heart sample were acquired on a Bruker Avance III 700 MHz NMR spectrometer (Bruker BioSpin, Germany) with a Bruker 4 mm TXI HR-MAS probe with z-gradients at 278 K and a spinning rate of 6 kHz using CPMG spin-echo pulse sequence. For all spectra of each heart sample, 128 transients were acquired with total T_2 filter time of 37.8 ms, 32 k data points, a spectral width of 14,097.744 Hz, and the relaxation delay of 4 s, and acquisition time of 1.162 s.

Free induction decays were weighted by an exponential function with a 0.3 Hz line-broadening factor prior to Fourier transformation. All acquired ^{1}H NMR spectra were phase- and baseline-corrected using TopSpin 3.1 software (Bruker BioSpin, Germany) and Chenomx NMR Suite Version 7.1 (Chenomx, Canada). The chemical shift was referenced to the proton signals of lactate at 1.32 ppm and formate at 8.45 ppm. Resonance assignments for serum and heart metabolites were accomplished using the 800 MHz library of Chenomx NMR Suite Version 7.1 (Chenomx, Canada) and literature reports[1-4]. A set of 2D NMR spectra (Fig. S7-9) was acquired on some representative serum and heart samples and spiked experiments were performed to confirm the metabolite identities. Representative 800 MHz ^{1}H NMR spectra of sera and 700 MHz HR- MAS ^{1}H NMR spectra of heart

tissues were shown in Fig. S10 and S11, respectively. The complete resonance assignment is listed in Table S3.

Concentration of 4,4-dimethyl-4-silapentane-1-sulfonic acid (DSS) was estimated using ERETIC2 (Electronic Reference To access In vivo Concentrations) module in TopSpin software 3.1 (Bruker BioSpin, Germany), which is based on the PULCON principle (PULse Length based CONcentrations determination)[5, 6]. Targeted metabolic profiling of serum and heart tissue were performed using Chenomx NMR Suite Version 7.1 (Chenomx, Canada) by integrating peak areas of metabolites compared with the areas of the DSS peak. For heart metabolites, tissue mass (g) was used to normalize metabolite levels.

Measurement of metabolites in sulfur amino acid metabolism

To determine concentration of sulfur amino acids, previously used methods were modified and adopted[7, 8]. In brief, to extract heart metabolites, 20 mg heart were transferred to each 1.5 mL microcentrifuge tube. 500 µL of 50 % aqueous methanol (v/v) containing 0.1 % formic acid and 0.05 % trifluoroacetic acid were added and vigorously mixed for 30 s. Homogenization with 2.8 mm zirconium oxide beads was performed at 5000 rpm twice using a Precellys 24 tissue grinder (Bertin Technologies, France). 250 µL of chloroform was added to each sample tube and mixed for 30 s. And then, the sample solution was incubated at 4 °C for 10 min and centrifuged at 4 °C and 13,000 rpm for 10 min. 400 µL of supernatant was transferred to new 1.5 mL tube and dried in a vacuum centrifuge. The dried sample was dissolved in 50 µL of 30 % aqueous methanol (v/v) containing 0.1 % formic acid, treated with a reducing agent of 50 µL of 500 mM dithiothreitol (DTT) solution resolved in 0.075 M NaOH to cleave disulphide bonds and then stored for 1 h at room temperature. To remove dust and particle in sample, purification was conducted using 0.2 µm PTFE membrane filter, the sample was transferred to LC glass vial and 500 ng/mL betaine-D_{11}, cysteine-D_2, methionine-D_3 and S-adenosylmethionine (SAM)-D_3 as internal standards were added to the vial. All these compounds were purchased from Cambridge Isotope Laboratories (MA, USA). 2 µL of sample was injected for each LC-MS/MS analysis.

To quantify the level of metabolites, liquid chromatography-mass spectrometry was performed on an Agilent 1290 Infinity LC and an Agilent 6490 Triple Quadrupole MS system equipped with Agilent Jet Stream ESI source (Agilent Technologies, USA). The MassHunter Workstation (Ver B.06.00, Agilent Technologies, USA) software was used for data acquisition and analysis. LC separations were carried out on Scherzo SM-C18 column (100 x 2 mm, particle size 3 µm, Imtakt, USA) on reverse phase mode for 7 min. Colum temperature and flow rate were set to 25 °C and 0.2 ml/min, respectively. The mobile phases used were 0.1 % formic acid in water (A) and methanol (B). The linear gradients were as follows: 0 % B for 1.5 min, 0-100 % B for 1.5 min, 100 % B for 0.5 min, 100-0 % B for 0.1 min, 0 % B for 3.4 min. MS/MS experiments were conducted on positive ion mode with following parameters: capillary voltage of 3.5 kV, nebulizer gas of nitrogen at 40 psi, drying gas temperature of 120 °C, drying gas flow rate of 11 L/min, sheath gas temperature of 400 °C, sheath gas flow rate of 12 L/min, nozzle voltage of 500 V. Quantification was performed in single reaction monitoring (SRM) mode and the optimized condition of each metabolite was performed using flow injection of individual standard compound solutions (100 ng/mL) into the mass. The SRM transitions, parameters on the *m/z* and collision energy of precursor and product ions are shown in Table S4.

LC-MS/MS-based lipidomic analysis

After extraction of sulfur amino acid metabolites in heart tissues, the solution under pellet was transferred to a 1.5-mL Eppendorf tube for lipidomic analysis of heart. Lipid extract of heart tissues was evaporated under a stream of nitrogen, diluted with an isopropanol: acetonitrile: water mixture (2:1:1, v/v/v), and transferred into vials after centrifugation for 10 min at 13,000g and 4°C.

To obtain MS spectral data, LC-ESI-MS/MS analyses of lipid extracts were performed on a triple TOF™ 5600 MS/MS System (AB Sciex, Canada) combined with a UPLC system (Waters, USA). LC separations were carried out on an Acquity UPLC BEH C18 column (100 x 2.1 mm, particle size 1.7 µm, Waters, USA). Colum temperature and flow rate were set to 35 °C and 0.35 ml/min, respectively. The mobile phases used were 10 mM ammonium acetate in an acetonitrile:

water mixture (40:60, v/v) (A) and acetonitrile: isopropanol mixture (10:90, v/v) (B). The linear gradients were as follows: 40–65% B for 5 min, 65-70% B for 7 min, 70-99% B for 3 min, 99% B for 2 min, 40% B for 3 min. The injection volume of the sample was 5 μL using partial loop mode for both positive and negative ionization polarity modes. Quality control (QC) samples, pooling identical aliquots of the samples, were measured regularly throughout the run for data reproducibility.

The spectral data were analyzed by MarkerView^{TM} (AB Sciex, Canada), which was used to find peaks, perform the alignment, and generate peak tables of *m/z* and retention times (min). Spectra were normalized to the median fold-change normalization. To identify reliable peaks and remove instrumental bias, peaks whose intensity in QC sample is lower than those in a blank sample were eliminated, and peaks with coefficients of variation below 20 in QC sample were selected. Lipids were tentatively identified by comparing the experimental data against various databases, including the METLIN (metlin.scripps.edu), Human Metabolome (www.hmdb.ca), MassBank (www.massbank.jp), and Lipid Maps (www.lipidmaps.org) databases. Fragment patterns (MS/MS spectra) were also used to identify lipid metabolites.

Real-time quantitative PCR primers

The qPCR was performed using the following primers: SCD1 sense (5′-TGC GAT ACA CTC TGG TGC TC-′) and antisense (5′-TAG TCG AAG GGG AAG GTG TG -3′), SREBP1 sense (5′-GCA GAC TCA CTG CTG CTG AC -3′) and antisense (5′-AGG TAC TGT GGC CAA GAT GG -3′), PPARα sense (5′-AAT GCA ATT CGC TTT GGA AG -′) and antisense (5′-GGC CTT GAC CTT GTT CAT GT -3′), PPARγ sense (5′-ACC TGA AGC TCC AAG AAT ACC A -3′) and antisense (5′-GAT GCT TTA TCC CCA CAG ACT C -3′), and β-actin sense (5′-TGT TAC CAA CTG GGA CGA CA-3′) and antisense (5′-GGG GTG TTG AAG GTC TCA AA-3′).

References

1. Beckonert, O. *et al*. High-resolution magic-angle-spinning NMR spectroscopy for metabolic profiling of intact tissues. *Nature protocols* **5**, 1019-1032 (2010).

2. Li, D. *et al*. Metabonomic changes associated with atherosclerosis progression for LDLR–/–mice. *Journal of proteome research* **14**, 2237-2254 (2015).

3. Jung, J. *et al*. Changes in one-carbon metabolism after duodenal-jejunal bypass surgery. *Am. J. Physiol. Endocrinol. Metab.* **310**, E624-632 (2016).

4. Jang, W. G. *et al*. Investigation of relative metabolic changes in the organs and plasma of rats exposed to X‐ ray radiation using HR‐ MAS 1H NMR and solution 1H NMR. *NMR Biomed.* **29**, 507-518 (2016).

5. Wider, G. & Dreier, L. Measuring protein concentrations by NMR spectroscopy. *J. Am. Chem. Soc.* **128**, 2571-2576 (2006).

6. Jung, Y., Hyeon, J. & Hwang, G. Software-assisted serum metabolite quantification using NMR. *Anal. Chim. Acta* **934**, 194-202 (2016).

7. Stevens, A. P. *et al*. Quantification of intermediates of the methionine and polyamine metabolism by liquid chromatography–tandem mass spectrometry in cultured tumor cells and liver biopsies. *Journal of chromatography A* **1217**, 3282-3288 (2010).

8. Van Liempd, S., Cabrera, D., Mato, J. & Falcon-Perez, J. A fast method for the quantitation of key metabolites of the methionine pathway in liver tissue by high-resolution mass spectrometry and hydrophilic interaction ultra-performance liquid chromatography. *Analytical and bioanalytical chemistry* **405**, 5301-5310 (2013).

Figure legends

Figure S1. PCA score plots obtained from NMR spectra of heart and serum.

PCA score plots of heart (a: R^2X = 70.4%, Q^2 = 39.8%) and serum (b: R^2X = 70.8%, Q^2 = 32.5%) at week 8. PCA score plots of heart (c: R^2X = 72.4%, Q^2 = 12.8%) and serum (d: R^2X = 67.1%, Q^2 = 14.7%) at week 16. PCA score plots of heart (e: R^2X = 81.2%, Q^2 = 27.6%) and serum (f: R^2X = 74.8%, Q^2 = 18.2%) at week 25.

Figure S2. Results of pathway analysis and correlation analysis.

(a) Overview of pathway analysis of heart tissue from AD and normal diet (ND) mice after 8 weeks. (b) Correlation analysis between heart metabolite and serum lipid levels from AD and ND mice after 8 weeks. (C) Overview of pathway analysis of heart tissue from AD and normal diet (ND) mice after 16 weeks. (d) Correlation analysis between heart metabolite and serum lipid levels from AD and ND mice after 16 weeks. The color and size of the circles reflect the p-values and pathway impact values, respectively. Spearman's correlation coefficients ranged in value from – 0.5 to + 0.5 and are indicated in red and blue for positive and negative correlations, respectively.

Figure S3. Representative extract ion chromatograms of betaine (a), betaine-D11 (b), cystathionine (c), cysteine (d), cysteine-D2 (e), dimethyl glycine (f), homocysteine (g), methionine (h), methionine-D3 (i), S-adenosylhomocysteine (SAH) (j), S-adenosylmethionine (SAM) (k), SAM-D3 (l), and serine (m) in heart tissue samples.

Figure S4. PCA score plots obtained from UPLC-QTOF MS spectra of heart lipid extracts.

PCA score plots in positive (a: R^2X = 65.5%, Q^2 = 52.0%) and negative mode (b: R^2X = 75.2%, Q^2 = 58.7%) at week 8. PCA score plots in positive (c: R^2X = 64.2%, Q^2 = 47.7%) and negative mode (d: R^2X = 74.5%, Q^2 = 49.8%) at week 16. PCA score plots in positive (e: R^2X = 63.5%, Q^2 = 48.5%) and negative mode (f: R^2X = 64.6%, Q^2 = 51.0%) at week 25.

Figure S5. PLS-DA score plots and permutation results obtained from the UPLC-QTOF MS spectra of heart lipid extracts. PLS-DA score plot (a: R^2X = 67.2%, R^2Y = 99.6%, Q^2 = 97.2%) and its validation plot (b) in positive ion mode. PLS-DA score plot (c: R^2X = 60.1%, R^2Y = 99.2%, Q^2 = 97.0%) and its validation plot (d) in negative ion mode. Validation plots were obtained from 100 permutation tests of the responses to the PLS-DA models.

Figure S6. Correlation analysis between FFAs and polar metabolites in heart tissues from mice fed ND and AD for 25 weeks (a). Spearman's correlation coefficients range in value from – 0.7 to + 0.7 and are indicated in red and blue for positive and negative correlation, respectively. The SCD1 desaturation index (b): the ratio of 16:1 and 16:0 or that of 18:1 and 18:0. *, **, and *** indicate $p < 0.05$, $p < 0.01$, $p < 0.001$, respectively.

Figure S7. Representative 2D ^{1}H-^{1}H TOCSY NMR spectrum of serum from mice fed an atherogenic diet for 25 weeks.

Figure S8. Representative 2D ^{1}H-^{13}C HSQC NMR spectrum of serum from mice fed an atherogenic diet for 25 weeks.

Figure S9. Representative 2D ^{1}H-^{13}C HSQC NMR spectrum of heart tissue from mice fed an atherogenic diet for 25 weeks.

Figure S10. Representative 800 MHz ^{1}H NMR spectra of sera from mice fed a normal diet for 8 weeks (a); an atherogenic diet for 8 weeks (b); a normal diet for 16 weeks (c); an atherogenic diet for 16 weeks (d); a normal diet for 25 weeks (e); an atherogenic diet for 25 weeks (f).

Key: 1, Leucine; 2, 2-Aminobutyrate; 3, Valine; 4, Isoleucine; 5, Isobutyrate; 6, 3-Hydroxybutyrate; 7, 2-Hydroxyisobutyrate; 8, Lactate; 9, Alanine; 10, Acetate; 11, Acetone; 12, Pyruvate; 13, Glutamine;

14, Citrate; 15, Methionine; 16, N,N-Dimethylglycine; 17, Creatine; 18, Lysine; 19, Creatinine; 20, O-Acetylcarnitine; 21, Choline; 22, Betaine; 23, Taurine; 24, Glycerol; 25, Glycine; 26, Glucose; 27, Glycolate; 28, Threonine; 29, Lactose; 30, Mannose; 31, Fumarate; 32, Tyrosine; 33, 2-phenylpropionate; 34, Phenylalanine; 35, Formate.

Figure S11. Representative 700 MHz HR- MAS ^{1}H NMR spectra of heart tissues from mice fed a normal diet for 8 weeks (a); an atherogenic diet for 8 weeks (b); a normal diet for 16 weeks (c); an atherogenic diet for 16 weeks (d); a normal diet for 25 weeks (e); an atherogenic diet for 25 weeks (f). Key: 1, Valine; 2, Isoleucine; 3, 3-Hydroxybutyrate; 4, Lactate; 5, Alanine; 6, Acetate; 7, Glutamate; 8, Glutamine; 9, Glutathione; 10, Methionine; 11, N,N-Dimethylglycine; 12, Creatine; 13, Lysine; 14, sn-Glycero-3-phosphocholine; 15, Choline; 16, Carnitine; 17, O-Phosphocholine; 18, Taurine; 19, Glycerol; 20, Glycine; 21, Glucose; 22, Threonine; 23, Inosine; 24, Nicotinurate.

Table legends

Table S1. List of lipid species selected by VIP and p-value with significantly different intensity between ND and AD mice after 25 weeks of feeding.

Table S2. Composition of the experimental diets (g/kg diet)

Table S3. ^{1}H and ^{13}C NMR peak assignments for identified metabolites.

Table S4. Performance parameters for characteristic transitions of UPLC-Triple Quadrupole MS used in the quantitative analysis of metabolites.

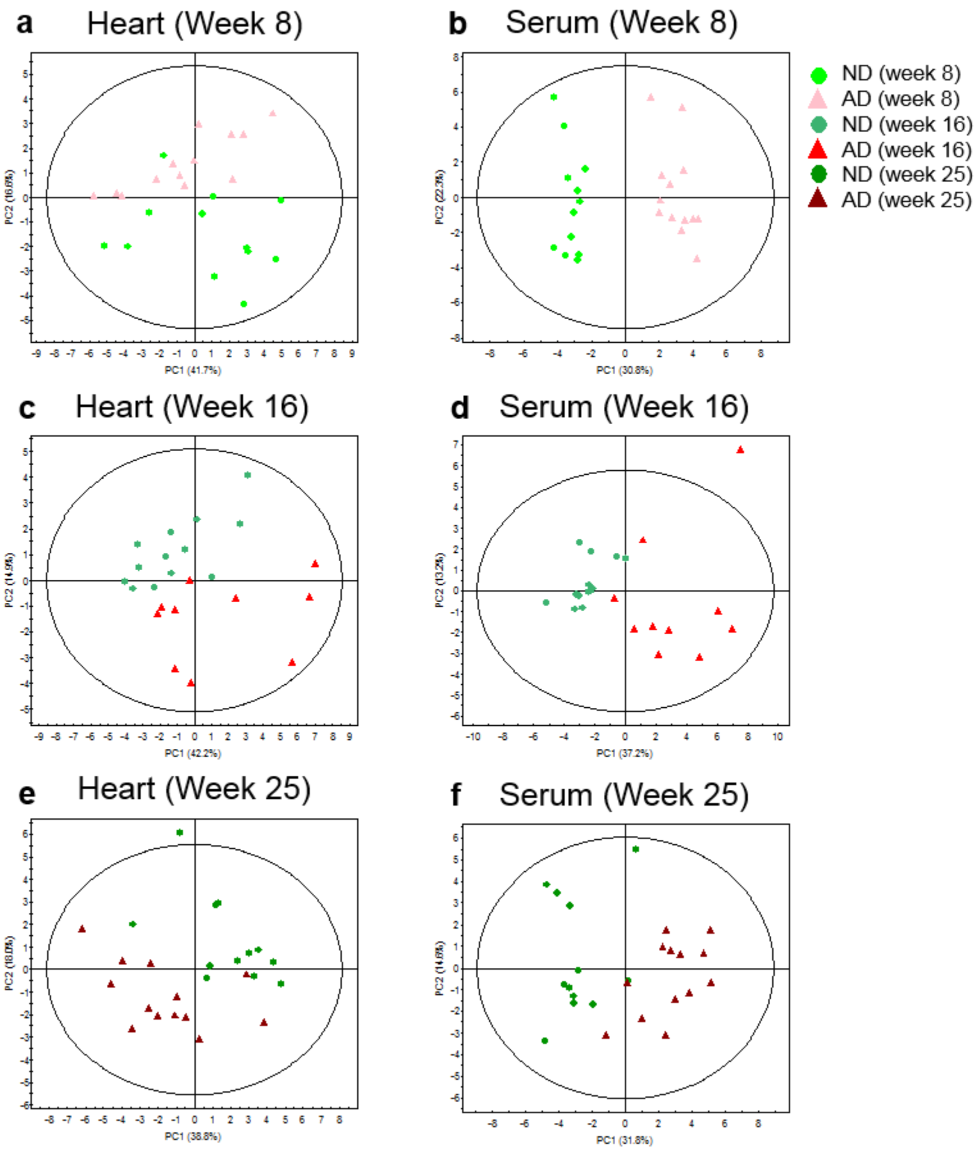

a Heart (Week 8)
b Serum (Week 8)
c Heart (Week 16)
d Serum (Week 16)
e Heart (Week 25)
f Serum (Week 25)
ND (week 8)
AD (week 8)
ND (week 16)
AD (week 16)
ND (week 25)
AD (week 25)
PC1 (41.7%)
PC1 (30.8%)
PC1 (42.2%)
PC1 (37.2%)
PC1 (38.8%)
PC1 (31.8%)

Figure S1.

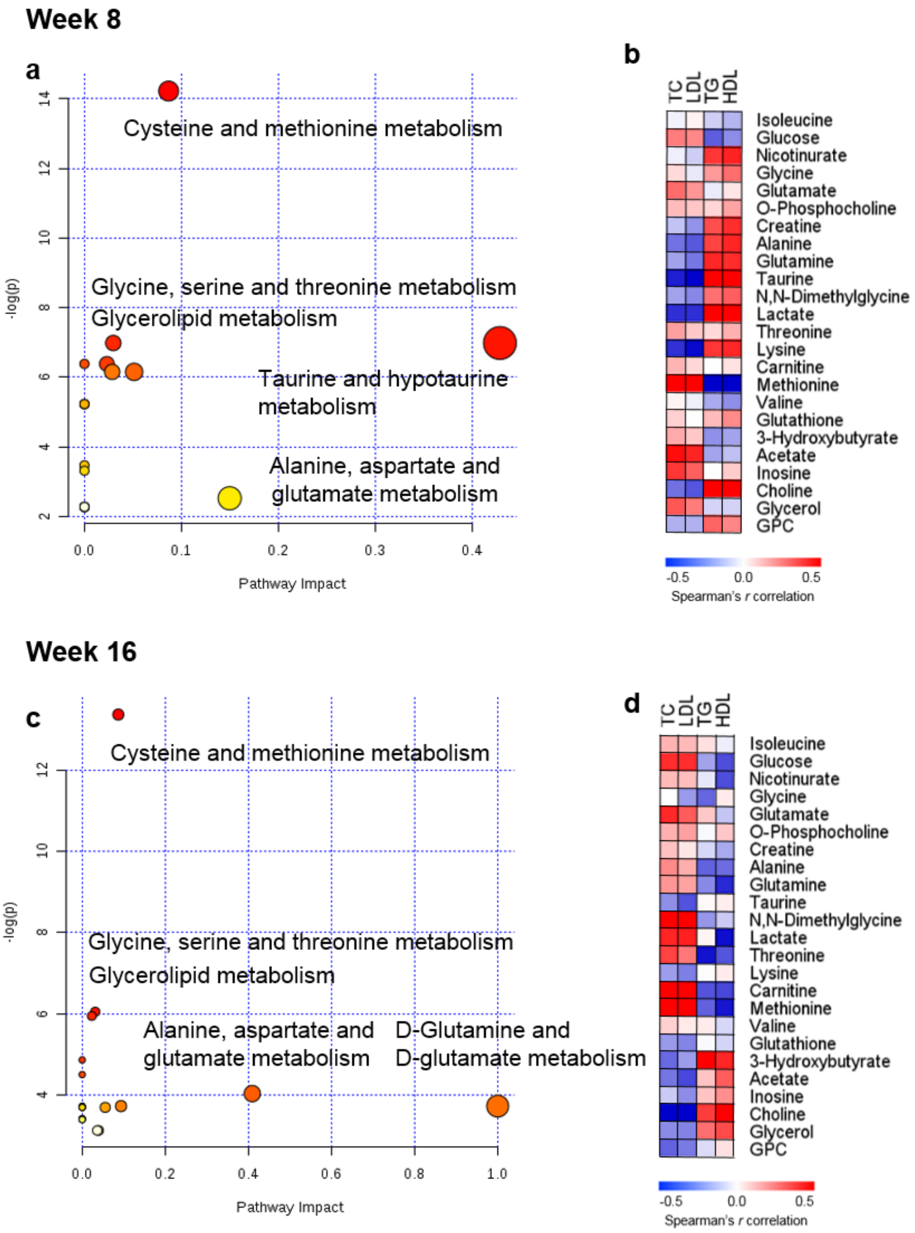

Week 8
a
Cysteine and methionine metabolism
Glycine, serine and threonine metabolism
Glycerolipid metabolism
Taurine and hypotaurine metabolism
Alanine, aspartate and glutamate metabolism
-log(p)
Pathway Impact
b
TC
LDL
TG
HDL
Isoleucine
Glucose
Nicotinurate
Glycine
Glutamate
O-Phosphocholine
Creatine
Alanine
Glutamine
Taurine
N,N-Dimethylglycine
Lactate
Threonine
Lysine
Carnitine
Methionine
Valine
Glutathione
3-Hydroxybutyrate
Acetate
Inosine
Choline
Glycerol
GPC
Spearman's r correlation
Week 16
c
Cysteine and methionine metabolism
Glycine, serine and threonine metabolism
Glycerolipid metabolism
Alanine, aspartate and glutamate metabolism
D-Glutamine and D-glutamate metabolism
-log(p)
Pathway Impact
d
TC
LDL
TG
HDL
Isoleucine
Glucose
Nicotinurate
Glycine
Glutamate
O-Phosphocholine
Creatine
Alanine
Glutamine
Taurine
N,N-Dimethylglycine
Lactate
Threonine
Lysine
Carnitine
Methionine
Valine
Glutathione
3-Hydroxybutyrate
Acetate
Inosine
Choline
Glycerol
GPC
Spearman's r correlation

Figure S2.

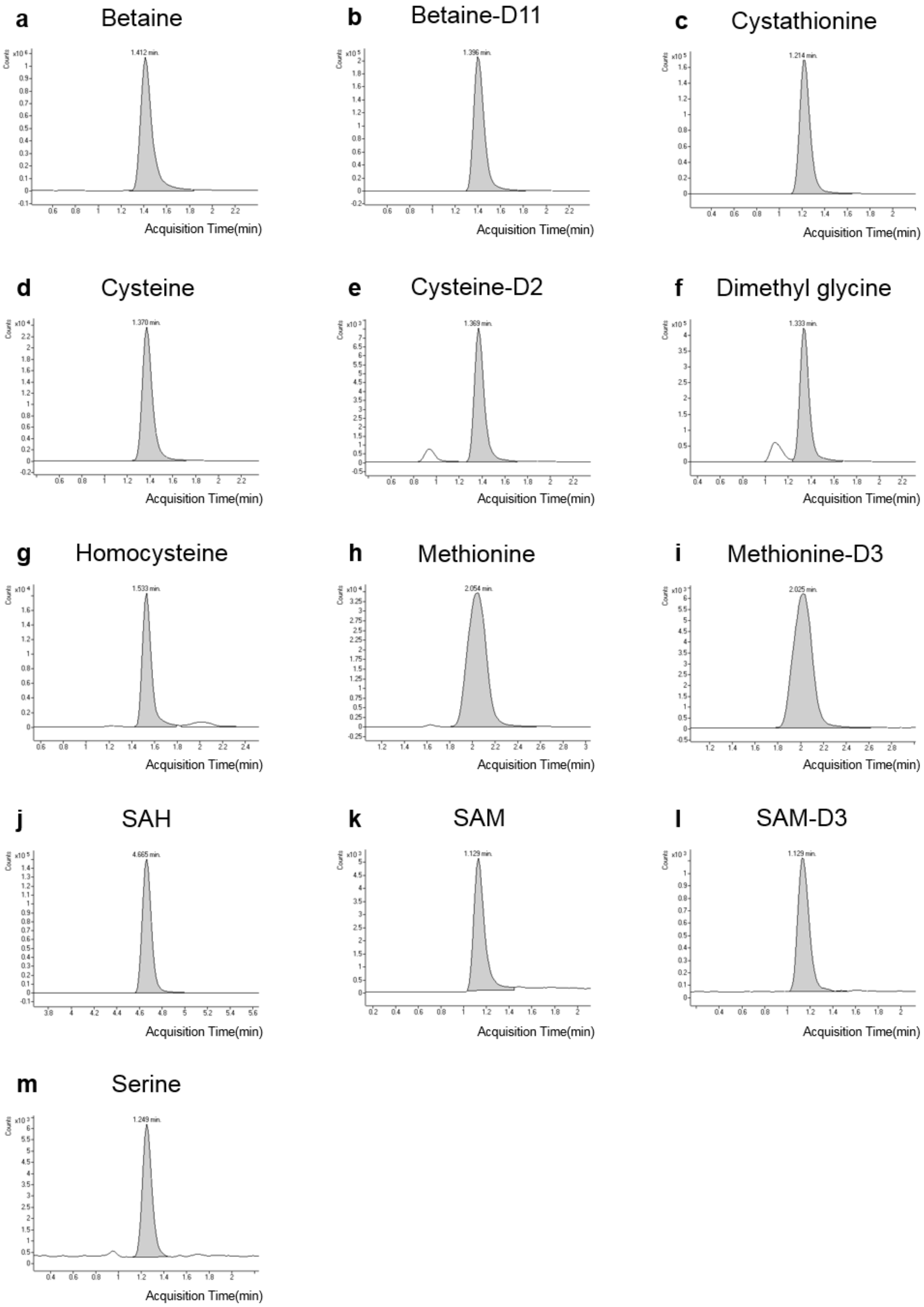
a
Betaine
b
Betaine-D11
c
Cystathionine
d
Cysteine
e
Cysteine-D2
f
Dimethyl glycine
g
Homocysteine
h
Methionine
i
Methionine-D3
j
SAH
k
SAM
l
SAM-D3
m
Serine
Counts
Acquisition Time(min)

Figure S3.

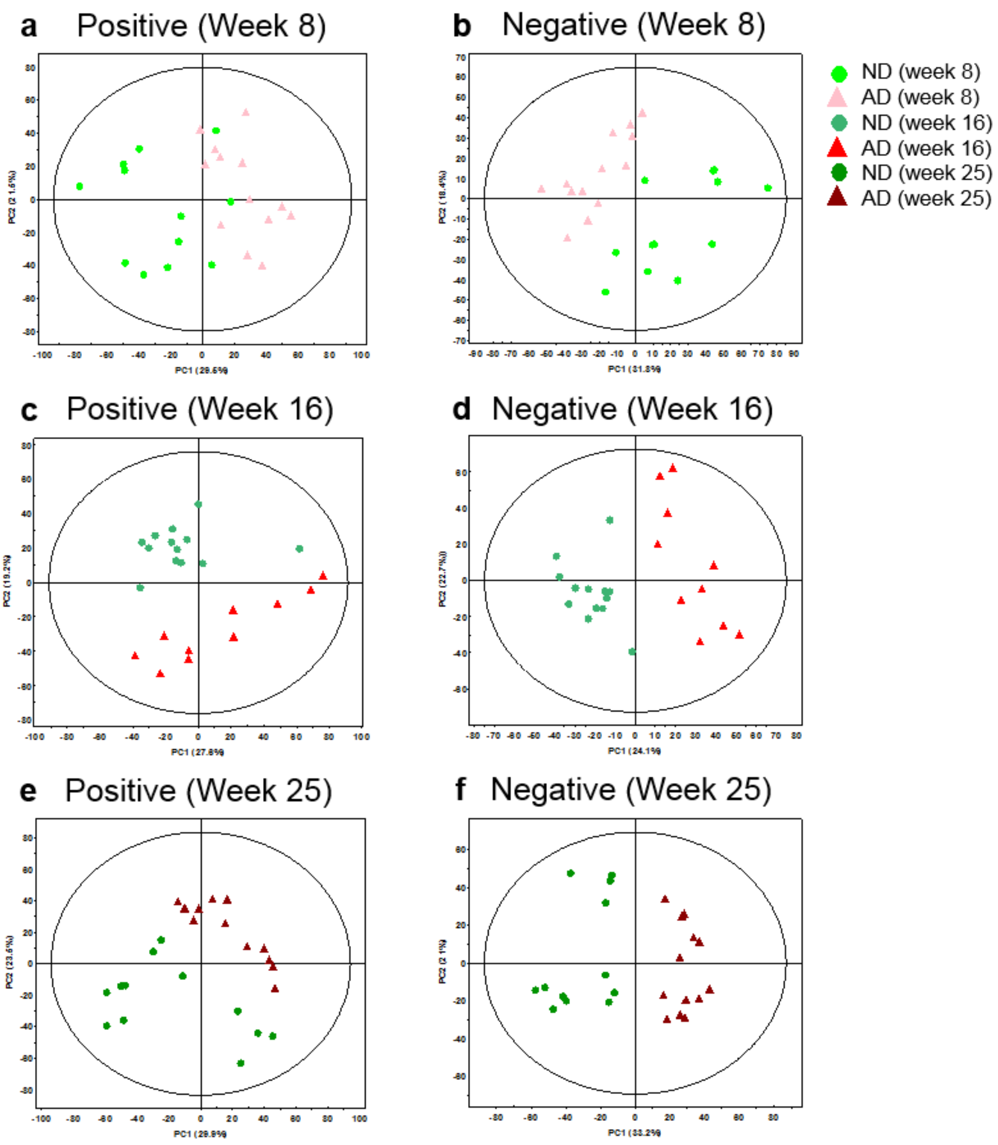
a Positive (Week 8)
b Negative (Week 8)
c Positive (Week 16)
d Negative (Week 16)
e Positive (Week 25)
f Negative (Week 25)
ND (week 8)
AD (week 8)
ND (week 16)
AD (week 16)
ND (week 25)
AD (week 25)

Figure S4.

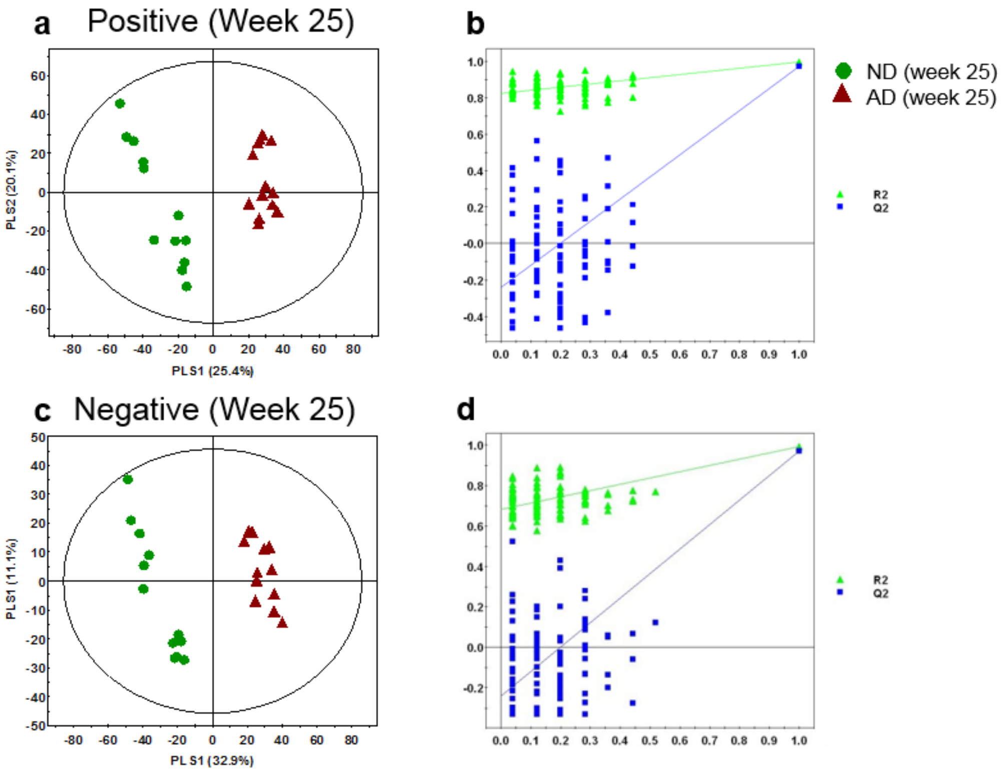
a
Positive (Week 25)
PLS2 (20.1%)
PLS1 (25.4%)
b
ND (week 25)
AD (week 25)
R2
Q2
c
Negative (Week 25)
PLS1 (11.1%)
PL S1 (32.9%)
d
R2
Q2

Figure S5.

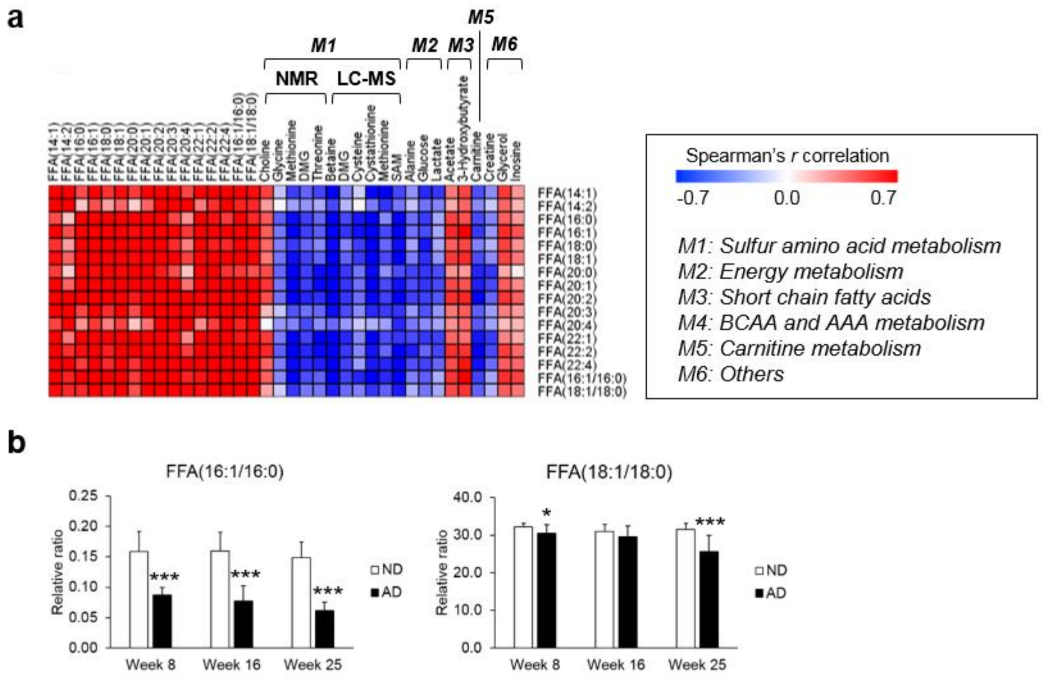
a
M1
M2
M3
M5
M6
NMR
LC-MS
Spearman's r correlation
-0.7
0.0
0.7
M1: Sulfur amino acid metabolism
M2: Energy metabolism
M3: Short chain fatty acids
M4: BCAA and AAA metabolism
M5: Carnitine metabolism
M6: Others
b
FFA(16:1/16:0)
FFA(18:1/18:0)
Relative ratio
Week 8
Week 16
Week 25
ND
AD

Figure S6.

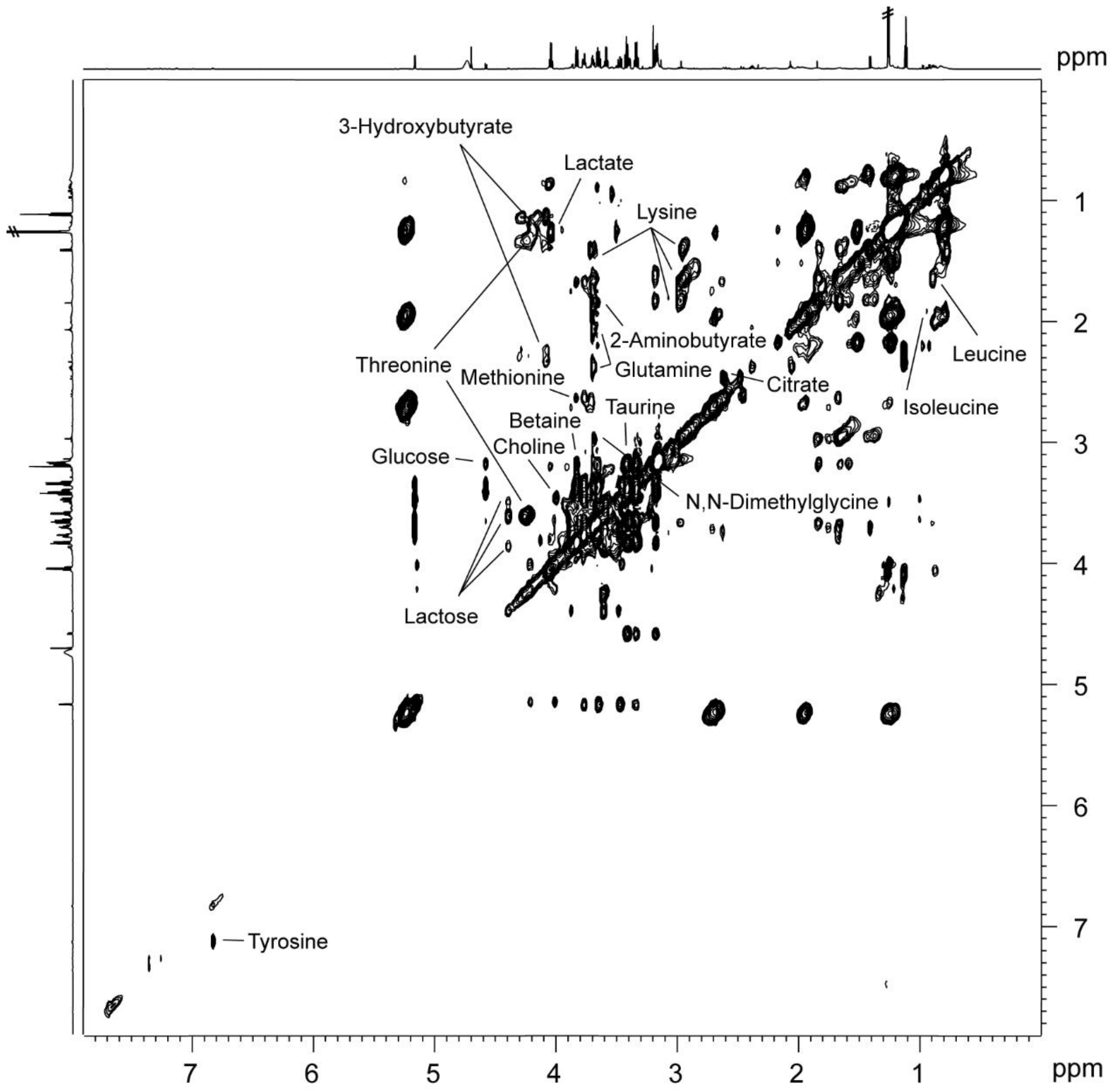
ppm
3-Hydroxybutyrate
Lactate
Lysine
Threonine
2-Aminobutyrate
Methionine
Glutamine
Citrate
Leucine
Isoleucine
Taurine
Betaine
Glucose
Choline
N,N-Dimethylglycine
Lactose
Tyrosine
7
6
5
4
3
2
1
ppm

Figure S7.

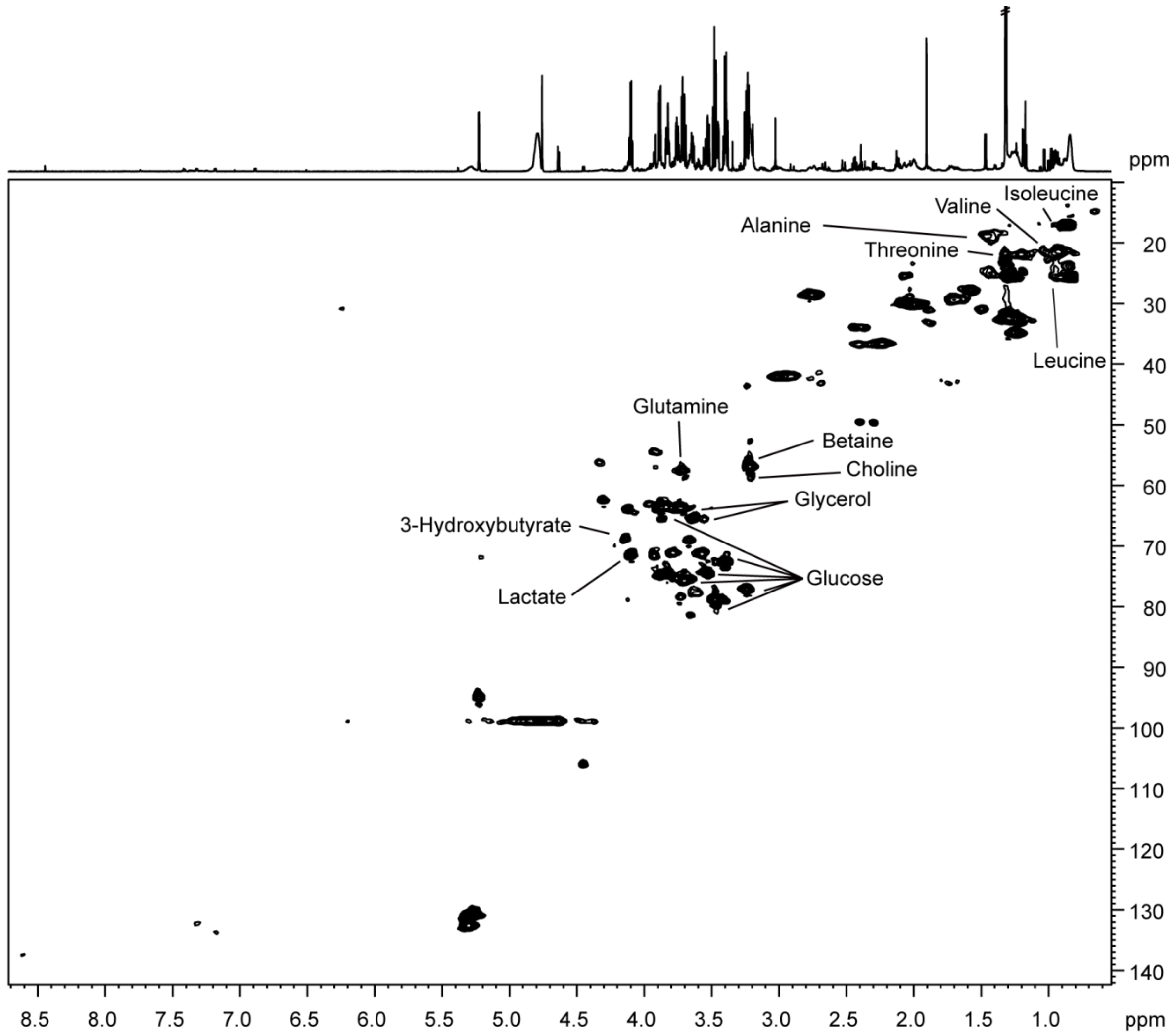
ppm
Isoleucine
Valine
Alanine
Threonine
Leucine
Glutamine
Betaine
Choline
Glycerol
3-Hydroxybutyrate
Glucose
Lactate
20
30
40
50
60
70
80
90
100
110
120
130
140
ppm
8.5
8.0
7.5
7.0
6.5
6.0
5.5
5.0
4.5
4.0
3.5
3.0
2.5
2.0
1.5
1.0
ppm

Figure S8.

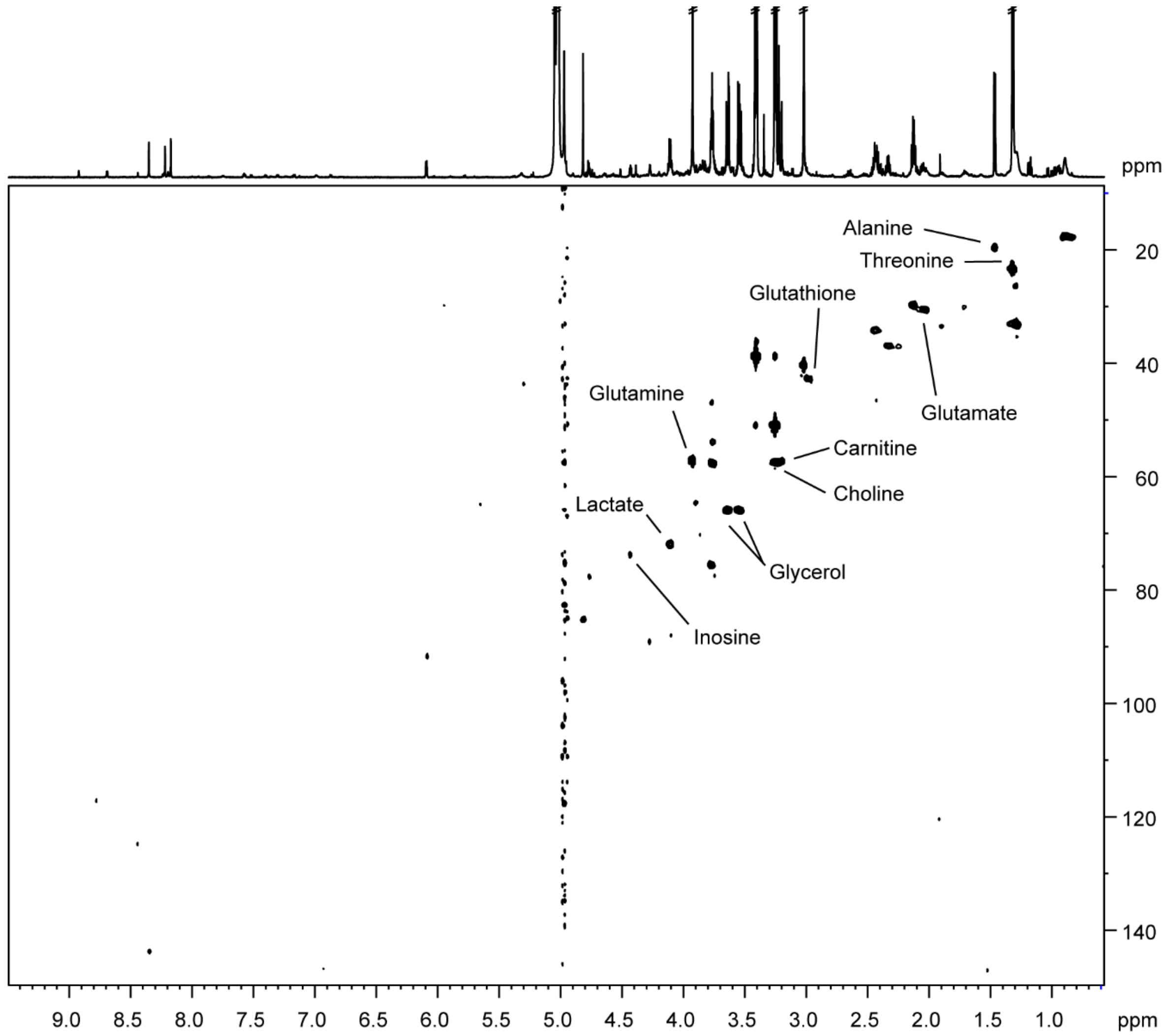

ppm
Alanine
Threonine
Glutathione
Glutamine
Glutamate
Carnitine
Choline
Lactate
Glycerol
Inosine
20
40
60
80
100
120
140
9.0 8.5 8.0 7.5 7.0 6.5 6.0 5.5 5.0 4.5 4.0 3.5 3.0 2.5 2.0 1.5 1.0
ppm

Figure S9.

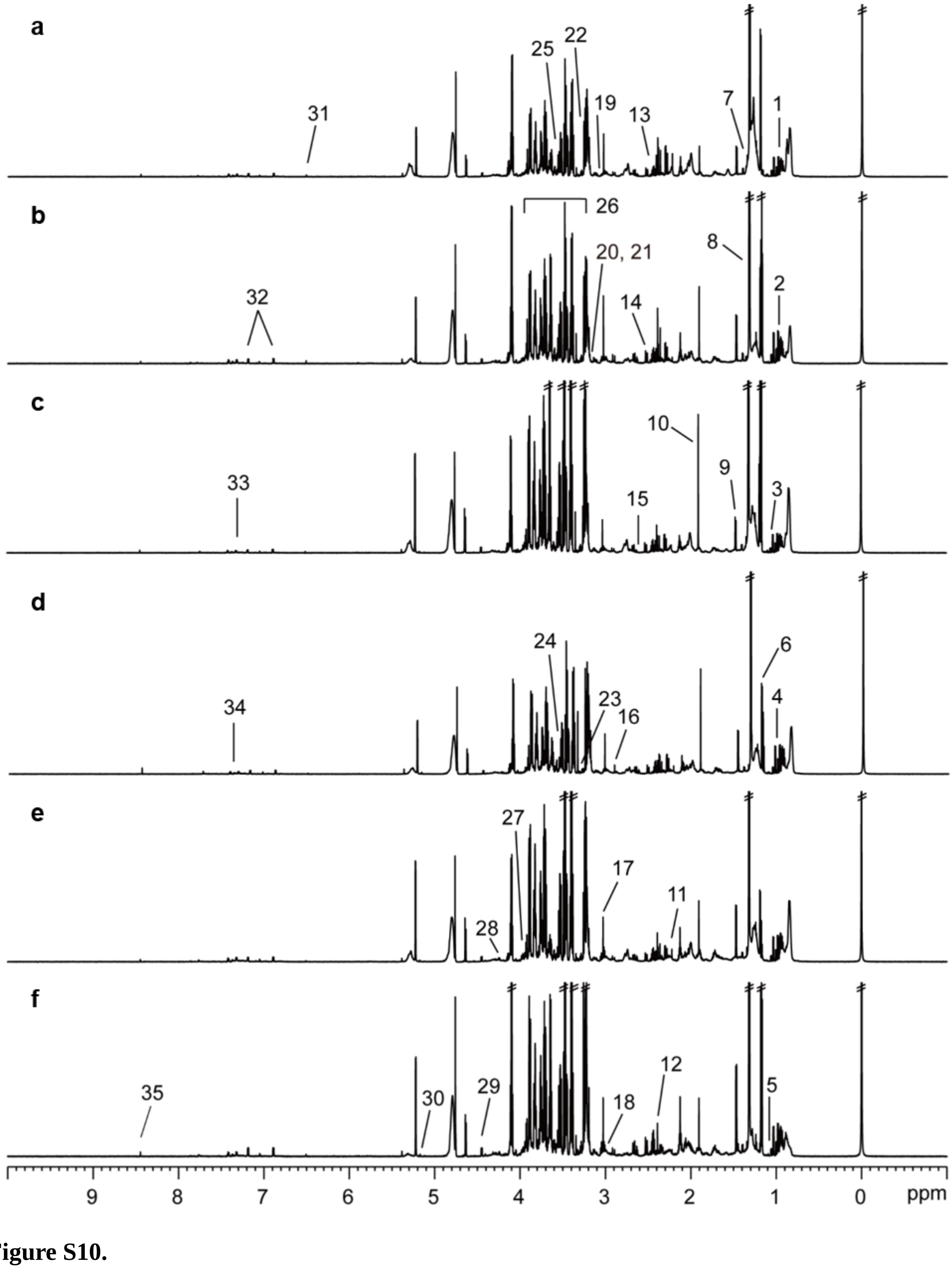
a
25
22
31
19
13
7
1
b
26
20, 21
8
32
14
2
c
10
9
33
15
3
d
24
6
23
34
16
4
e
27
17
11
28
f
12
35
29
30
18
5
9
8
7
6
5
4
3
2
1
0
ppm

Figure S10.

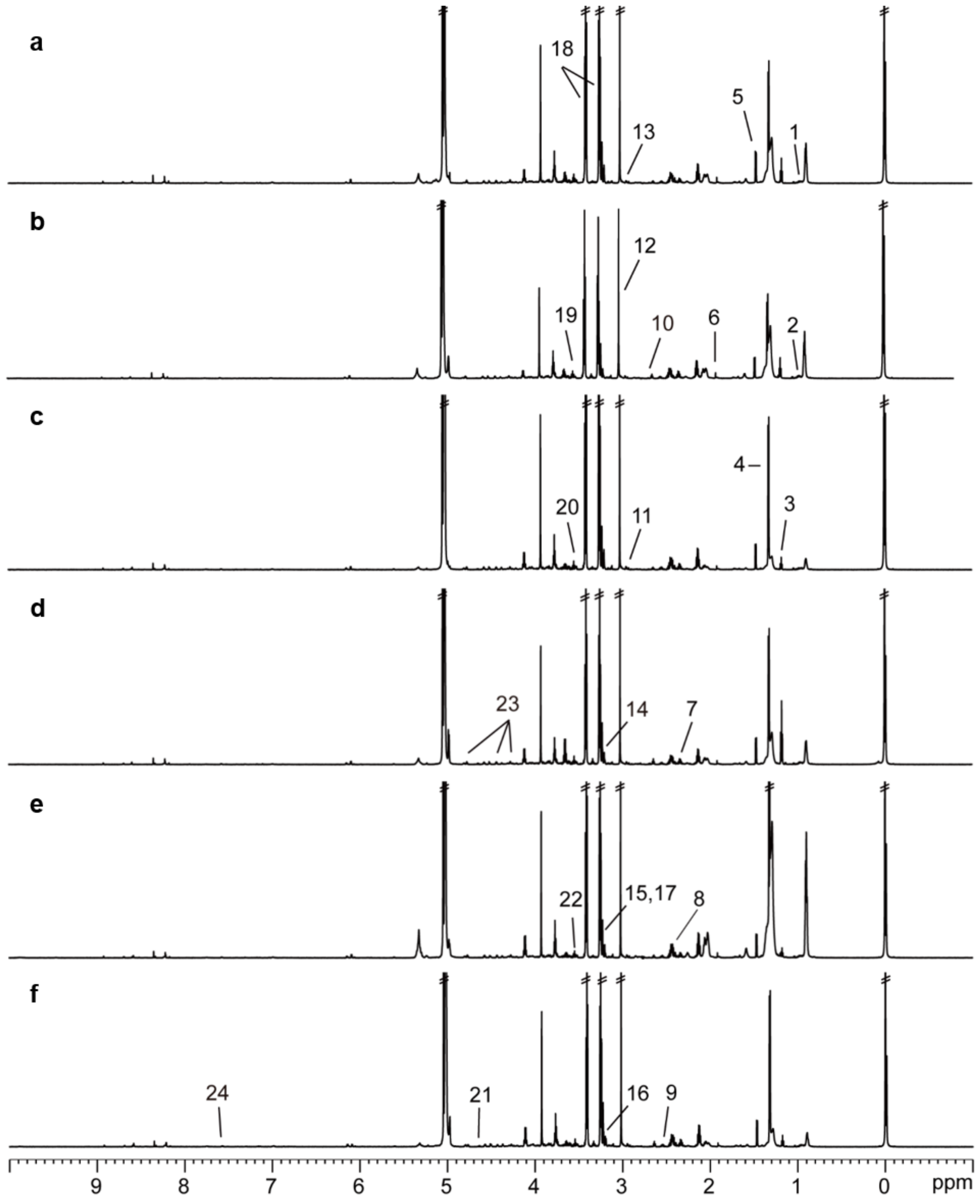
a
18
5
13
1
b
12
19
10
6
2
c
4
20
11
3
d
23
14
7
e
22
15,17
8
f
24
21
16
9
9
8
7
6
5
4
3
2
1
0
ppm

Figure S11.

Table S1. List of lipid species selected by VIP and p-value with significantly different intensity between ND and AD mice after 25 weeks of feeding.

Metabolite	*m/z*	Ion species	Ret. Time (min)	CV (% RSD)	p-Value[a]			Fold Change (AS/ND)			VIP[b]
					Week 8	Week 16	Week 25	Week 8	Week 16	Week 25	Week 25
Cer (d18:1/16:0)	538.5113	[M+H]+	11.01	4.5	0.077	0.784	0.000	0.84	0.97	0.61	1.49
Cer (d18:1/19:0)	578.5442	[M-H]-	13.74	4.9	0.001	0.001	0.000	0.69	0.53	0.47	1.43
Cer (d18:1/20:0)	594.5746	[M+H]+	14.17	2.7	0.186	0.376	0.001	0.87	0.88	0.66	1.27
Cer (d18:1/23:0)	636.6144	[M+H]+	14.81	4.1	0.979	0.563	0.005	0.96	0.90	0.79	1.10
Cer (d18:1/24:1)	648.6220	[M+H]+	14.58	1.9	0.002	0.057	0.000	0.80	0.88	0.72	1.53
Cer (d18:2/16:0)	594.4995	[M+CH3COO]-	9.2	8.3	0.000	0.000	0.000	0.61	0.60	0.49	1.40
Cer (d18:2/20:0)	592.5580	[M+H]+	13.32	4.5	0.270	0.030	0.000	0.87	0.80	0.66	1.44
Cer (d18:2/21:0)	664.5722	[M+CH3COO]-	13.74	7.8	0.000	0.000	0.000	0.41	0.34	0.27	1.63
Cer (d18:2/23:0)	634.5998	[M+H]+	14.45	5.4	0.137	0.042	0.000	0.88	0.84	0.71	1.48
DG (34:1)	612.5467	[M+NH4]+	13.85	6.1	0.000	0.976	0.007	0.72	1.00	0.66	1.09
DG (36:0)	642.5966	[M+NH4]+	14.85	10.3	0.001	0.012	0.000	1.60	1.80	1.81	1.40
DG (38:3)	664.5742	[M+NH4]+	13.99	2.3	0.000	0.000	0.000	0.50	0.51	0.43	1.73
DG (38:5)	643.5162	[M+H]+	13.89	6.3	0.060	0.522	0.014	0.79	1.18	0.58	1.09
DG (40:3)	692.6158	[M+NH4]+	14.5	6.9	0.000	0.000	0.000	0.57	0.41	0.44	1.62
DG (40:6)	686.5611	[M+NH4]+	13.62	3.1	0.002	0.000	0.003	1.90	2.69	2.17	1.31
DG (42:2)	722.6504	[M+NH4]+	15.05	6.2	0.168	0.003	0.022	1.10	0.65	0.77	1.00
FFA (14:1)	225.185	[M-H]-	1.74	2.0	0.002	0.101	0.000	0.72	0.75	0.54	1.28
FFA (14:2)	223.1688	[M-H]-	1.38	5.8	0.002	0.186	0.022	0.67	0.83	0.66	1.18
FFA (16:0)	255.2325	[M-H]-	3.22	2.0	0.068	0.343	0.000	0.90	0.96	0.76	1.30
FFA (16:1)	253.2164	[M-H]-	2.4	1.8	0.000	0.000	0.000	0.49	0.47	0.32	1.57
FFA (18:0)	283.2527	[M-H]-	3.41	2.6	0.077	0.343	0.000	0.84	0.95	0.65	1.30
FFA (18:1)	281.2456	[M-H]-	3.41	2.4	0.040	0.376	0.000	0.80	0.90	0.53	1.39
FFA (20:0)	311.2942	[M-H]-	5.47	8.4	0.060	0.000	0.000	0.81	0.70	0.68	1.13
FFA (20:1)	309.2761	[M-H]-	4.5	3.0	0.000	0.000	0.000	0.53	0.33	0.25	1.61
FFA (20:2)	307.2621	[M-H]-	3.63	5.0	0.000	0.000	0.000	0.54	0.43	0.29	1.57

FFA (20:3)	305.2466	[M-H]-	2.98	4.4	0.046	0.003	0.001	0.58	0.62	0.45	1.26
FFA (20:4)	303.2299	[M-H]-	2.52	3.2	0.068	0.088	0.026	0.59	0.74	0.45	1.05
FFA (22:1)	337.3078	[M-H]-	5.56	6.1	0.003	0.000	0.000	0.66	0.37	0.40	1.43
FFA (22:2)	335.2932	[M-H]-	4.78	3.5	0.000	0.000	0.000	0.48	0.32	0.28	1.56
FFA (22:4)	333.2763	[M-H]-	4.11	2.8	0.000	0.000	0.000	0.17	0.13	0.11	1.56
GlcCer (d18:1/22:0)	784.6518	[M+H]+	14.32	1.6	0.000	0.000	0.000	1.50	1.89	2.13	1.41
GlcCer (d18:1/23:0)	798.6672	[M+H]+	14.53	10.0	0.001	0.000	0.000	1.30	1.49	1.54	1.04
LysoPC (14:0)	468.3054	[M+H]+	1.87	6.2	0.000	0.000	0.000	2.30	1.90	1.66	1.35
LysoPC (16:0)	496.3378	[M+H]+	2.95	2.6	0.538	0.879	0.016	0.93	0.96	0.76	1.02
LysoPC (16:1)	494.3194	[M+H]+	2.16	4.0	0.000	0.000	0.000	0.49	0.47	0.44	1.56
LysoPC (18:1)	522.3542	[M+H]+	3.08	4.3	0.000	0.015	0.000	0.68	0.72	0.55	1.41
LysoPC (20:1)	550.3786	[M+H]+	4.09	4.8	0.000	0.000	0.000	0.32	0.35	0.31	1.71
LysoPC (20:2)	548.3679	[M+H]+	3.28	6.9	0.000	0.000	0.000	0.42	0.56	0.55	1.58
LysoPC (20:4)	544.3360	[M+H]+	2.16	2.2	0.005	0.131	0.030	0.52	0.74	0.55	1.15
LysoPC (22:2)	593.4153	[M+NH4]+	4.53	6.1	0.003	0.000	0.000	1.27	1.34	1.35	1.73
LysoPC (22:4)	572.3657	[M+H]+	2.81	2.5	0.000	0.000	0.000	0.19	0.23	0.19	1.69
LysoPC (22:5)	570.3528	[M+H]+	2.54	1.6	0.000	0.000	0.000	0.11	0.14	0.07	1.57
LysoPC (28:0)	664.5155	[M+H]+	8.45	8.1	0.000	0.000	0.000	7.77	6.09	6.75	1.69
LysoPC (O-18:0)	510.3853	[M+H]+	4.55	6.0	0.000	0.000	0.000	3.24	2.16	1.95	1.81
LysoPE (17:0)	466.2841	[M-H]-	3.58	8.7	0.004	0.002	0.000	0.66	0.62	0.46	1.35
LysoPE (18:1)	478.2875	[M-H]-	3.18	3.1	0.000	0.012	0.002	0.60	0.68	0.57	1.19
LysoPE (22:4)	530.3202	[M+H]+	2.97	4.1	0.000	0.000	0.000	0.40	0.45	0.45	1.57
LysoPE (22:5)	526.2798	[M-H]-	2.65	5.0	0.000	0.000	0.000	0.14	0.16	0.11	1.40
LysoPE (22:6)	524.2744	[M-H]-	2.12	1.6	0.186	0.148	0.022	1.40	2.12	1.99	1.01
PC (22:4)	630.3642	[M+CH3COO]-	2.79	11.5	0.000	0.000	0.000	0.37	0.44	0.44	1.44
PC (24:0)	608.4325	[M+H]+	4.05	12.7	0.000	0.000	0.000	0.60	0.61	0.65	1.27
PC (28:0)	678.4979	[M+H]+	7.57	2.9	0.000	0.000	0.000	4.35	7.87	9.34	1.55
PC (30:0)	706.5236	[M+H]+	9.14	2.6	0.000	0.000	0.000	2.22	2.15	2.44	1.85
PC (32:0)	734.5520	[M+H]+	11.03	2.8	0.000	0.000	0.000	0.64	0.64	0.62	1.88
PC (32:2)	730.5299	[M+H]+	7.98	2.7	0.000	0.002	0.000	1.44	1.71	2.24	1.34
PC (34:0)	762.5809	[M+H]+	13.16	4.5	0.007	0.000	0.000	1.13	1.22	1.29	1.59
PC (34:3)	756.5441	[M+H]+	11.01	4.1	0.000	0.000	0.000	0.74	0.68	0.70	1.70
PC (34:5)	752.5063	[M+H]+	3.38	11.7	0.005	0.018	0.016	1.50	1.38	1.50	1.06
PC (36:2)	786.5844	[M+H]+	11.58	3.5	0.000	0.000	0.000	1.62	2.02	1.95	1.70
PC (36:5)	780.5422	[M+H]+	7.89	3.6	0.001	0.003	0.040	0.41	0.57	0.62	1.03

PC (36:7)	776.5414	[M+H]+	12.1	3.8	0.003	0.000	0.001	1.67	2.45	2.50	1.45
PC (38:0)	818.6457	[M+H]+	14.04	4.6	0.000	0.042	0.000	0.49	0.75	0.57	1.39
PC (38:1)	816.6287	[M+H]+	14.08	1.6	0.000	0.000	0.000	0.68	0.65	0.72	1.83
PC (38:3)	870.608	[M+CH3COO]-	12.05	3.7	0.137	0.446	0.019	1.13	1.11	1.39	1.02
PC (38:5)	830.5536	[M+Na]+	10.11	4.2	0.000	0.000	0.000	0.21	0.22	0.17	1.64
PC (38:6)	806.5538	[M+H]+	8.94	4.4	0.225	0.030	0.026	1.35	2.55	1.98	1.11
PC (38:9)	817.5394	[M+NH4]+	8.95	13.1	0.186	0.036	0.019	1.38	2.47	2.11	1.12
PC (40:2)	842.6480	[M+H]+	14.04	3.6	0.000	0.000	0.000	0.51	0.56	0.63	1.84
PC (40:4)	838.6186	[M+H]+	12.78	2.8	0.000	0.000	0.000	0.31	0.42	0.43	1.51
PC (40:5)	836.6027	[M+H]+	12.11	3.3	0.000	0.000	0.000	0.17	0.20	0.13	1.60
PC (40:6)	834.5847	[M+H]+	10.74	1.5	0.022	0.005	0.014	1.68	3.16	2.18	1.18
PC (40:7)	832.5693	[M+H]+	8.97	1.5	0.002	0.006	0.022	0.35	0.52	0.38	1.23
PC (40:8)	830.5547	[M+H]+	7.81	3.6	0.406	0.148	0.030	1.14	2.53	1.85	1.02
PC (42:5)	864.6320	[M+H]+	13.73	6.3	0.000	0.000	0.000	0.22	0.21	0.16	1.68
PC (42:6)	862.6202	[M+H]+	12.05	3.5	0.000	0.000	0.000	0.25	0.31	0.26	1.54
PC (42:9)	856.5724	[M+H]+	8.45	6.5	0.000	0.000	0.040	0.34	0.37	0.40	1.29
PC (44:10)	882.6101	[M+H]+	8.36	6.8	0.000	0.000	0.011	0.60	0.53	0.62	1.15
PC (44:11)	880.6053	[M+H]+	6.58	6.6	0.008	0.042	0.004	1.68	1.74	1.78	1.19
PC (P-15:0)	466.3238	[M+H]+	4.64	2.7	0.001	0.000	0.002	1.49	1.83	1.57	1.35
PC(P-18:0/22:5)	820.6444	[M+H]+	13.13	5.8	0.152	0.376	0.000	1.08	1.07	1.27	1.42
PE (34:0)	720.5425	[M+H]+	13.48	5.5	0.376	0.067	0.000	1.03	1.17	1.39	1.44
PE (34:1)	718.5289	[M+H]+	11.59	3.1	0.000	0.000	0.000	0.68	0.69	0.78	1.67
PE (36:1)	746.5528	[M+H]+	13.5	2.2	0.247	0.042	0.000	1.07	1.12	1.49	1.72
PE (36:2)	744.5394	[M+H]+	11.98	2.5	0.005	0.042	0.000	1.21	1.19	1.51	1.73
PE (38:1)	772.5767	[M-H]-	14.19	8.0	0.000	0.000	0.000	0.46	0.39	0.61	1.46
PE (38:4)	768.5342	[M+H]+	11.69	2.2	0.728	0.101	0.004	1.00	1.32	1.60	1.25
PE (38:5)	764.5186	[M-H]-	10.33	2.2	0.000	0.000	0.000	0.15	0.15	0.15	1.42
PE (38:6)	764.5059	[M+H]+	9.28	6.3	0.406	0.131	0.030	1.14	1.63	1.62	1.01
PE (40:5)	792.5483	[M-H]-	12.42	6.8	0.000	0.000	0.000	0.17	0.16	0.17	1.39
PE (40:7)	790.5202	[M+H]+	11.75	4.8	0.936	0.376	0.002	1.01	1.10	1.48	1.31
PE (P-18:0/22:6)	774.5327	[M-H]-	12	4.6	0.005	0.001	0.001	1.91	2.81	3.33	1.25
PE (P-18:1/18:1)	728.5454	[M+H]+	13.06	6.3	0.001	0.000	0.000	1.40	1.63	2.06	1.65
SM (d18:1/16:0)	703.5611	[M+H]+	9.25	5.5	0.001	0.000	0.000	1.21	1.41	1.39	1.45
SM (d18:1/20:0)	781.5974	[M+Na]+	13.3	5.1	0.016	0.042	0.000	1.10	1.15	1.21	1.45
SM (d18:1/21:0)	773.6371	[M+H]+	13.84	3.3	0.002	0.000	0.005	0.84	0.77	0.83	1.19

SM (d18:1/24:0)	815.6815	[M+H]+	14.64	1.6	0.000	0.000	0.000	1.35	1.37	1.54	1.63
SM (d18:2/18:0)	729.5746	[M+H]+	9.42	4.7	0.000	0.000	0.000	1.75	2.10	1.97	1.80
SM (d18:2/23:0)	799.6565	[M+H]+	13.89	7.3	0.000	0.000	0.019	0.81	0.78	0.87	1.04
TG (42:1)	738.6503	[M+NH4]+	15.08	7.6	0.002	0.000	0.005	1.88	1.85	1.61	1.19
TG (44:1)	766.6796	[M+NH4]+	15.3	3.7	0.019	0.001	0.007	1.47	1.72	1.51	1.15
TG (44:2)	764.6633	[M+NH4]+	15.11	5.2	0.077	0.001	0.010	1.48	1.69	1.44	1.05
TG (46:1)	794.7142	[M+NH4]+	15.47	2.4	0.014	0.001	0.001	1.44	1.84	1.85	1.34
TG (46:2)	792.6983	[M+NH4]+	15.31	2.8	0.022	0.000	0.002	1.45	1.92	1.84	1.31
TG (48:3)	818.7125	[M+NH4]+	15.3	2.9	0.040	0.002	0.001	1.51	2.36	2.28	1.34
TG (56:3)	930.8293	[M+NH4]+	15.83	5.7	0.077	0.257	0.004	0.60	0.76	0.49	1.24
TG (56:4)	928.8157	[M+NH4]+	15.71	3.8	0.016	0.186	0.002	0.54	0.73	0.45	1.34
TG (56:6)	924.7814	[M+NH4]+	15.54	3.5	0.014	0.927	0.040	0.54	1.03	0.53	1.06
TG (56:7)	905.7443	[M+H]+	15.6	6.0	0.503	0.376	0.046	0.83	1.53	0.65	1.09
TG (58:6)	952.8161	[M+NH4]+	15.64	5.9	0.000	0.166	0.000	0.39	0.62	0.36	1.32
TG (58:8)	948.7769	[M+NH4]+	14.65	3.1	0.005	0.410	0.035	0.55	2.14	0.45	1.01

[a] Mann–Whitney *U*-test was performed to calculate p-value.

[b] Variable importance in the projection (VIP) was derived from PLS-DA models.

Table S2. Composition of the experimental diets (g/kg diet)

Ingredients	Groups	
	Normal diet	Atherogenic diet
Corn starch	540	275
Casein	200	75
Sucrose	100	30
Corn oil	60	—
Soybean oil	—	50
Mineral mix	35	35
Vitamin mix	10	10
Cellulose	50	90
DL-Methionine	3	2
Choline bitartrate	2	2
Cocoa butter	—	75
Cholesterol	—	12.5
BHT	0.01	—
Soy protein	—	130
Maltodextrin	—	150
Calcium carbonate	—	5.5
Potassium citrate	—	10
Sodium cholic acid	—	5
Total	1000	954

Table S3. ^{1}H and ^{13}C NMR peak assignments for identified metabolites.

Metabolites	δ^{1}H (multiplicity)[a]	δ^{13}C	Identification method	Biomatrices[b]
Leucine	0.95 (d)	25.50	1D, spiking, TOCSY, HSQC	S
	**0.96 (d)**	25.50		
	1.71 (m)	43.1		
	3.73 (dd)	53.9		
2-Aminobutyrate	**0.97 (t)**		1D, spiking, TOCSY	S
	1.89 (m)			
	3.71 (m)			
Valine	**0.98 (d)**		1D, spiking, HSQC	H, S
	1.03 (d)	21.01		
	2.27 (m)			
	3.60 (d)			
Isoleucine	0.93 (d)	17.05	1D, spiking, TOCSY, HSQC	H, S
	**1.00 (t)**			
	1.26 (m)	25.47		
	1.46 (m)			
	1.98 (m)	39.2		
	3.66 (d)			
Isobutyrate	**1.06(d)**		1D, spiking,	S
	2.38(m)			
3-Hydroxybutyrate	**1.19 (d)**	24.7	1D, spiking, TOCSY, HSQC	H, S
	2.29 (dd)	49.5		
	2.40 (dd)	49.5		
	4.14 (m)	68.7		
2-Hydroxyisobutyrate	**1.3 (s)**		1D, spiking	S
Lactate	**1.32 (d)**	23.5	1D, spiking, TOCSY, HSQC	H, S
	4.10 (q)	71.4		
Alanine	1.47 (d)	19.6	1D, spiking, HSQC	H, S
	3.78 (q)	53.3		
Acetate	**1.90 (s)**		1D, spiking	H, S
Acetone	**2.22 (s)**		1D, spiking	S
Glutamate	2.04 (m)	30.3	1D, spiking, HSQC	H
	2.12 (m)	30.1		
	2.32 (m)	36.6		
	**2.35 (m)**	36.6		
	3.75 (m)	57.4		
Pyruvate	**2.36 (s)**		1D, spiking	S
Glutamine	2.12 (m)	29.70	1D, spiking, TOCSY, HSQC	H, S
	2.14 (m)	29.70		

	2.43 (m)	36.4		
	**2.46 (m)**	33.84		
	3.77 (t)	57.30		
	6.86 (br)			
	7.60 (br)			
Citrate	**2.52 (d)**		1D, spiking, TOCSY	S
	2.67 (d)			
Glutathione	2.15 (m)		1D, spiking, HSQC	H
	2.17 (m)			
	2.53 (m)			
	**2.56 (m)**			
	2.93 (m)			
	2.98 (m)	42.6		
	3.76 (d)	57.6		
	3.77 (t)	57.6		
	3.78 (d)	57.6		
	4.57 (m)			
	8.24 (m)			
	8.50 (s)			
Methionine	2.11 (m)		1D, spiking, TOCSY	H, S
	2.13 (s)			
	2.19 (m)			
	**2.63 (t)**			
	3.85 (dd)			
N,N-Dimethylglycine	**2.92 (s)**		1D, spiking, TOCSY	H, S
	3.72 (s)			
Creatine	**3.02** (s)		1D, spiking	H, S
	3.92 (s)			
Lysine	1.43 (m)		1D, spiking, TOCSY	H, S
	1.50 (m)			
	1.71 (m)			
	1.89 (m)			
	**3.02 (t)**			
	3.74 (t)			
Creatinine	**3.03** (s)	32.97	1D, spiking	S
	4.05 (s)	59.16		
O-Acetylcarnitine	2.13 (s)		1D, spiking	S
	2.50 (dd)			
	2.63 (dd)			
	**3.19 (s)**			
	3.59 (d)			

	3.84 (dd)			
	5.89 (m)			
sn-Glycero-3-phosphocholine	**3.20 (s)**		1D, spiking	H
	3.60 (m)			
	3.67 (m)			
	3.67 (dd)			
	3.87 (m)			
	3.91 (m)			
	3.94 (m)			
	4.31 (m)			
Choline	**3.20 (s)**	58.03	1D, spiking, TOCSY, HSQC	H, S
	3.51 (m)			
	4.06 (m)	58.21		
Carnitine	2.41 (m)	46.5	1D, spiking, HSQC	H
	2.45 (m)	46.5		
	**3.22 (s)**	57.4		
	3.40 (m)			
	3.43 (m)			
	4.57 (m)			
O-Phosphocholine	**3.22 (s)**		1D, spiking	H
	3.58 (m)			
	4.16 (m)			
Betaine	**3.26 (s)**	56.82	1D, spiking, TOCSY, HSQC	S
	3.89 (s)	69.3		
Taurine	3.25 (t)		1D, spiking, TOCSY	H, S
	**3.42 (t)**			
Glycerol	**3.55 (dd)**	65.39	1D, spiking, HSQC	H, S
	3.64 (dd)	65.39		
	3.77 (m)	71.07		
Glycine	**3.55 (s)**		1D, spiking	H, S
Glucose	3.23 (dd)	77.15	1D, spiking, TOCSY, HSQC	H, S
	3.39 (t)	72.48		
	3.4 (t)	72.48		
	3.46 (m)	78.94		
	3.48 (t)	78.94		
	3.53 (dd)	74.19		
	3.70 (t)	63.77		
	3.71 (dd)	63.77		
	3.75 (dd)	63.77		
	3.82 (m)	63.59		

	3.83 (dd)	63.59		
	3.89 (dd)	63.59		
	**4.64 (d)**	100.03		
	5.22 (d)	94.99		
Glycolate	**3.93 (s)**		1D, spiking	S
Threonine	1.32 (d)	22.2	1D, spiking, TOCSY, HSQC	H, S
	**3.58 (d)**	63.6		
	4.25 (m)			
Inosine	3.83 (m)	64.4	1D, spiking, HSQC	H
	3.90 (m)	64.4		
	4.27 (m)			
	**4.43 (m)**	73.6		
	4.77 (t)	77.5		
	6.10 (d)	91.6		
	8.22 (s)			
	8.35 (s)	143.9		
Lactose	3.28 (t)		1D, spiking, TOCSY	S
	3.54 (dd)			
	3.55 (dd)			
	3.58 (dd)			
	3.60 (m)			
	3.65 (m)			
	3.72 (m)			
	3.75 (m)			
	3.79 (m)			
	3.83 (t)			
	3.85 (dd)			
	3.88 (dd)			
	3.94 (m)			
	**4.45 (dd)**			
	4.66 (d)			
	5.22 (d)			
Mannose	3.37 (m)		1D, spiking	S
	3.57 (t)			
	3.65 (m)			
	3.72 (dd)			
	3.76 (dd)			
	3.80 (m)			
	3.84 (dd)			
	3.87 (dd)			
	3.89 (dd)			

	3.93 (m)		
	4.89 (d)		
	**5.17 (d)**		
Fumarate	**6.01 (s)**	1D, spiking	S
Tyrosine	3.05 (dd)	1D, spiking, TOCSY	S
	3.19 (dd)		
	3.93 (dd)		
	6.89 (d)		
	**7.19 (d)**		
2-Phenylpropionate	1.40(d)	1D, spiking	S
	3.64(m)		
	7.29(m)		
	7.34(m)		
	**7.38(m)**		
Phenylalanine	3.12 (dd)	1D, spiking	S
	3.28 (dd)		
	3.99 (dd)		
	7.32 (d)		
	7.37 (t)		
	**7.42 (t)**		
Nicotinurate	3.97 (d)	1D, spiking	H
	**7.57 (m)**		
	8.23 (m)		
	8.69 (m)		
	8.92 (d)		
Formate	**8.45** (s)	1D, spiking	S

[a] Letters in parentheses mean the peak multiplicities: s, singlet; d, doublet; t, triplet; dd, doublet of doublet; q, quartet; m, multiplet. [b] Letters in parentheses mean the metabolites were detected in heart (H) and serum (S). Bold letters mean ^{1}H signal that was used to calculate the integral area.

Table S4. Performance parameters for characteristic transitions of UPLC/Triple Quadrupole MS used in the quantitative analysis of metabolites.

Metabolites	Q1	Q3	DP (V)	CE (eV)	CXP (V)
Betaine	118.1	58.1	380	36	5
Betaine-D_{11}	129.0	66.0	380	22	5
Cystathionine	223.1	87.9	380	28	5
Cysteine	122.0	59.0	380	26	5
Cysteine-D_2	124.0	61.0	380	14	5
Dimethyl glycine	104.1	58.1	380	12	5
Homocysteine	135.9	89.8	380	8	5
Methionine	149.9	56.0	380	14	5
Methionine-D_3	153.0	56.0	380	14	5
SAH	385.0	136.1	380	20	5
SAM	399.2	250.1	380	12	5
SAM-D_3	402.0	250.1	380	12	5
Serine	106.0	88.0	380	6	5
